# COVID-19 and COVID-19 vaccination experiences and perceptions and their predictors among community members during the COVID-19 pandemic in Ebonyi state, Nigeria: an analytical cross-sectional study

**DOI:** 10.1186/s12889-024-18028-5

**Published:** 2024-02-22

**Authors:** Ugwu I. Omale, Victor U. Uduma, Azuka S. Adeke, Cordis O. Ikegwuonu, Glory E. Nkwo, Ugochi IA. Nwali, Olaedo O. Nnachi, Okechukwu O. Ukpabi, Ifeyinwa M. Okeke, Richard L. Ewah, Osarhiemen Iyare, Onyinyechukwu U. Oka, Chidinma I. Amuzie

**Affiliations:** 1 Department of Community Medicine, Alex Ekwueme Federal University Teaching Hospital Abakaliki (AEFUTHA), Abakaliki, Ebonyi State, Nigeria; 2 Department of Internal Medicine, Alex Ekwueme Federal University Teaching Hospital Abakaliki (AEFUTHA), Abakaliki, Ebonyi State Nigeria; 3 Department of Community Medicine, Federal Medical Centre Umuahia, Umuahia, Abia State Nigeria; 4 Department of Anaesthesia, Alex Ekwueme Federal University Teaching Hospital Abakaliki (AEFUTHA), Abakaliki, Ebonyi State, Nigeria; 5 Anaesthesia Unit, Department of Surgery, Ebonyi State University, Abakaliki, Ebonyi State, Nigeria

**Keywords:** COVID-19, COVID-19 vaccination/vaccination process, Experiences, Perceptions, Expectations, Community members, Nigeria

## Abstract

**Background:**

COVID-19 is still a disease of global public health importance which requires long term application of control measures as millions of new infections or re-infections and thousands of related deaths still occur worldwide and the risk of an upsurge from new strains of the virus continues to be a threat. The decrease in the use of and non-use of preventive public health measures are among the factors fuelling the disease. The (previous) experiences and perceptions of people regarding the COVID-19 pandemic, COVID-19 vaccination, and the vaccination process are factors that will influence subsequent use of preventive/control measures. We explored the COVID-19 and COVID-19 vaccination and the vaccination process experiences and perceptions, and their predictors, among the community members in Ebonyi state, Nigeria.

**Methods:**

We conducted an analytical cross-sectional study between March 12 and May 9, 2022 among all consenting/assenting community members aged 15 years and above in 28 randomly selected geographical clusters. A structured interviewer-administered electronic questionnaire in KoBoCollect installed in android devices was used to collect data which was analysed using descriptive statistics and bivariate and multivariate generalized estimating equations.

**Results:**

Of the 10,825 community members surveyed: only 31.6% had strong COVID-19 experience and perception, 72.2% had good COVID-19 vaccination expectation and perception, and only 54.2% had positive COVID-19 vaccination process experience and perception. The most important predictors of the extent/level of COVID-19 and COVID-19 vaccination and the vaccination process experiences and perceptions were level of attitude towards COVID-19 and COVID-19 vaccination and level of knowledge about COVID-19. Other important predictors were marital status, educational level, and main occupation.

**Conclusions:**

This study’s evidence, including the identified predictors, will inform subsequent policy actions regarding COVID-19 in the strategies to improve the COVID-19 and COVID-19 vaccination and the vaccination process experiences and perceptions of community members (and their use of preventive/control measures) in Ebonyi state and Nigeria, and other similar contexts. It will also inform future policy actions/strategies regarding similar diseases.

**Supplementary Information:**

The online version contains supplementary material available at 10.1186/s12889-024-18028-5.

## Background

The coronavirus disease 2019 (COVID-19) caused an unprecedented pandemic and has been affecting the lives and livelihood of the world population since 2019 (for the past four years) [1]. Even though it has been declared to no longer be a public health emergency of international concern, [2] COVID-19 is still a disease of global public health importance that requires long term application of control measures as millions of new infections or re-infections and thousands of related deaths still occur worldwide and the risk of an upsurge from new strains of the severe acute respiratory syndrome coronavirus 2 (SARS-CoV-2) continues to be a threat [1, 3]. Although the rate of testing and reporting have decreased (and testing and reporting have ceased in some countries) [1, 3], more than 1.1 million COVID-19 cases and 8700 related deaths were confirmed worldwide between 11 December 2023 and 7 January 2024, with more than 3300 cases in Africa [3]. 

The decrease in the use of and non-use of preventive public health measures, including COVID-19 vaccination, are among the factors fuelling the global threat of the disease [1]. Disease risk perception, confidence in vaccination (in terms of safety/side-effects and effectiveness) and the vaccination process (in terms of ease of access and the appeal of the vaccination system) are factors that influence the acceptance of vaccination as a preventive measure against diseases [4]. COVID-19 vaccination (and other preventive measures) is one of the strategies for the long-term management and control of the disease [1]. The (previous) experiences and perceptions of people regarding the COVID-19 pandemic, COVID-19 vaccination, and the vaccination process are factors that will very likely influence subsequent acceptance/uptake of COVID-19 vaccination. COVID-19 experiences and perceptions would, in addition to influencing the acceptance/uptake of COVID-19 vaccination, [5, 6] also influence the subsequent use of other preventive measures in situations of any COVID-19 upsurge from new variants of the virus [7]. 

Based on our observations during the pandemic, many people in Ebonyi state and Nigeria strongly believed they had gotten COVID-19, or that they knew other persons who had gotten COVID-19, based only on the symptoms they experienced (without laboratory tests). Whether there were confirmatory laboratory tests or not, such belief or perceptions would influence their practices/behaviours regarding the pandemic. Even in instances of laboratory confirmed cases of COVID-19 and related deaths, the belief or perception that such cases and deaths were actually due to COVID-19 or not would be an important factor that influence healthy behaviours. Such belief or perception is not uncommon in Ebonyi state (and Nigeria/other African countries) where morbidity and mortality related to many common diseases (with known causes based on scientific knowledge) are often being attributed to superstitious causes (such as spiritual attacks). This was perhaps more pronounced during the COVID-19 pandemic, as we observed anecdotally, due to the misinformation, disinformation and conspiracy theories about COVID-19 and COVID-19 vaccination that overwhelmed the conventional and social media.

An understanding of people’s COVID-19 experiences and perceptions, and the determinants, would be important in the subsequent planning of tailored COVID-19 behaviour change communication strategies in Ebonyi state and other similar settings. There was therefore the need to assess and explore people’s experiences and perceptions of the COVID-19 pandemic and COVID-19 vaccination and the vaccination process in Ebonyi state in order to provide a more complete picture.

We carried out an extensive geographical community-based study to assess COVID-19 vaccination acceptance and the determinants among the community members in Ebonyi state [8]. As part of the study, we also assessed and explored the experiences and perceptions about COVID-19 and COVID-19 vaccination and its processes, and their predictors, among the community members during the pandemic in Ebonyi state, Nigeria, in order to generate evidence to inform subsequent policy actions and interventions regarding the global threats from COVID-19 and other possible outbreaks of similar diseases in the future.

## Methods

### Study design and participants

The study was an analytical cross-sectional survey which was conducted between March 12 and May 9, 2022 among community members in geographical clusters in Ebonyi state, southeast of Nigeria. The study protocol has been described elsewhere [8]. Clusters were the immediate catchment communities/villages of primary healthcare (PHC) facilities and eligible clusters were those with 200 or more households, or a population of 1000 or more people, whose PHC facilities were providing basic maternal and child healthcare services including routine childhood immunization, that could be easily accessed with a car, and where the cluster heads gave verbal consents. Eligible community members were those aged 15 years and above who gave verbal consent or assent. Eligible participants were selected by stratified cluster sampling technique. The list of clusters obtained from the Ebonyi State Ministry of Health was used to prepare the list of eligible clusters as the sampling frame. Random samples of 21 and 7 clusters were respectively selected from the rural and urban/semi-urban strata using the “sample” command in Stata. In the selected clusters, all the households were visited and the eligible household members were selected to participate in the survey. A sample size of 28 clusters with 15,032 community members was estimated for the parent study [8] and 28 clusters and 10,825 (72.0%) community members successfully participated in the survey.

### Data collection

Data was collected through population-based household survey using a structured interviewer-administered community members questionnaire. The questionnaire design was informed by published data and expert validation and pre-tests were carried out by the research team [8]. The questionnaire had several sections including sociodemographic characteristics; COVID-19 experiences and perceptions; basic knowledge of COVID-19; and attitude towards COVID-19 and COVID-19 vaccination. The electronic version of the questionnaire was programmed using the KoBoToolbox software and was pre-tested in non-participating clusters. Trained interviewers administered the electronic questionnaire with KoBoCollect installed in android phones or tablet devices. All eligible household members were interviewed. Completed questionnaires were uploaded daily and reviewed for missing, incoherent, and illogical data. The data management and quality control is described in greater details in the study protocol [8]. 

### Independent factors and outcome measures

The independent factors were sociodemographic characteristics, main and most trusted sources of information about COVID-19, level of knowledge of COVID-19, and level of attitude towards COVID-19 and COVID-19 vaccination. The basic knowledge of COVID-19 was assessed using 44 knowledge items, each scored “1” for correct response and “0” for incorrect response with the highest attainable score of 44 and lowest of zero for each participant. Knowledge score of ≥ 75% of 44 was good knowledge and < 75% was poor knowledge. The attitude towards COVID-19 and COVID-19 vaccination was assessed using 16 attitude items, each on a five-category scale of strongly disagree, disagree, not sure, agree, and strongly agree and scored from “1” to “5” or “5” to “1” as appropriate. For each participant, the highest attainable attitude score was 80 and the lowest was 16. Attitude score of ≥ 75% of 80 was good attitude and < 75% was poor attitude.

The main outcome measures were the extent of COVID-19 experience and perception, level of COVID-19 vaccination expectation and perception, and level of COVID-19 vaccination process experience and perception. These outcomes were measured by using 5–8 questionnaire items to assess the experiences and perceptions of participants about COVID-19 and COVID-19 vaccination and the vaccination process, scoring the five categories of each item from 0 to 4, summing the scores for the 5–8 items related to each outcome for each participant, and grading the total scores on a two-level scale such that scores ≥ 50% of the total versus < 50% were respectively considered to be: strong versus not strong COVID-19 experience and perception; good versus poor COVID-19 vaccination expectation and perception; and positive versus negative COVID-19 vaccination process experience and perception. Greater details are described in the study protocol [8]. 

“Experience and perception” was explored as particular outcomes/variables because “experience” and “perception” are naturally interrelated and influence each other. For example, in the context of COVID-19, someone who have witnessed a case of COVID-19 in the locality might be more likely to perceive/believe that COVID-19 is real and that it is possible to get infected. Conversely, someone who perceive/believe that COVID-19 is real and that it is possible to get infected, will be more likely to experience (observe facts of) COVID-19 cases in the locality. In other words, someone who perceive/believe that COVID-19 is not real and that it is not possible to get infected, will be more likely not to experience (not to observe facts of) COVID-19 cases in the locality because any case of COVID-19 in the locality (base on classical symptoms and or lab tests) can more easily be interpreted to be other diseases or “spiritual attack”.

The other outcomes were the COVID-19 and COVID-19 vaccination and the vaccination process experiences and perceptions which were assessed with the five-category 5–8 questionnaire items and dichotomized into positive and non-positive category variables like: fear of getting COVID-19 (very fearful/a little fearful versus not fearful at all/not fearful/not sure), fear of having severe side-effects from COVID-19 vaccination (not fearful at all/not fearful versus very fearful/a little fearful/not sure), etc.

### Statistical analyses

Statistical analyses were done with Stata/SE version 15.1 (Stata Corp, College Station, TX, USA). Data was summarized using descriptive statistics, frequencies with proportions (expressed as percentages) and median with inter-quartile range as appropriate. Inferential statistics were done using population-averaged models to account for clustering and at 2.5% significance level to correct for multiple comparisons. For dichotomous or categorical independent factors, prevalence difference in the outcomes with 97.5% CI and *p*-values were computed using binomial identity generalized estimating equations (GEE) with an exchangeable correlation matrix and robust standard errors. For continuous independent factors, coefficients in the outcomes with 97.5% CI and *p*-values were computed using the binomial identity GEE models. All the independent factors were added to the GEE model in the adjusted analyses. For the binomial identity GEE models that failed to achieve convergence, gaussian identity GEE models were used instead [9]. 

## Results

### Sociodemographic characteristics

The sociodemographic characteristics of the 10,825 community members who participated in the study are presented in Table 1. The median age of participants (IQR) was 30 years (21–45). Majority were females (56.1%), were married (52.8%), had a secondary education (56.2%) were self-employed (54.6%), and were living in rural areas (77.7%) and more of them (41.6%) had usual monthly income of 20,000 Nigerian naira (NGN) or less.

### COVID-19 and COVID-19 vaccination and the vaccination process experiences and perceptions

The COVID-19 and COVID-19 vaccination and the vaccination process experiences and perceptions of the 10,825 community members who participated in the study are presented in Table 2 Regarding COVID-19 experiences and perceptions, more of the participants were very fearful about getting COVID-19 (25.6%) and had the perception that it was not possible at all for them to get COVID-19 (27.6%). Majority of them were sure they had never gotten COVID-19 (61.4%) and did not know any person who had gotten COVID-19 (90.5%).

**Table 1:** Sociodemographic characteristics of the 10,825 study participants

	n	%
Gender		
Male	4749	43.9
Female	6076	56.1
Age, median (IQR), years	30 (21–45)	–
Marital status		
Married	5712	52.8
Not married*	5113	47.2
Educational level		
No formal education	1065	9.8
Primary	2211	20.4
Secondary	6083	56.2
Tertiary	1466	13.5
Main occupation		
Self-employment^	5907	54.6
Private paid work	720	6.6
Government paid work	636	5.9
Others^^	3562	32.9
Residence		
Urban/semi-urban	2409	22.3
Rural	8416	77.7
Usual monthly income, NGN		
No income	2980	27.5
20,000 and less	4500	41.6
More than 20,000	3345	30.9

*Separated or Divorced or Widowed or Never married (Single). ^Farmer or Trader or Other self-employments. ^^Housewife or Student or Apprentice or Youth Corper or None. NGN = Nigerian naira

Regarding COVID-19 vaccination expectations and perceptions, more of the participants had the perception that it was important for them to receive COVID-19 vaccination (30.2%), were not sure about their level of fear of severe side-effects from COVID-19 vaccination (22.9%), and believed COVID-19 vaccination would give them full protection against COVID-19 (36.3%). Regarding COVID-19 vaccination process experiences and perceptions, majority of the participants said they had heard many times that COVID-19 vaccination was available for them to go and receive (57.0%) and more of them said they did not know any COVID-19 vaccination place/site (41.9%).

**Table 2:** COVID-19 and COVID-19 vaccination and the vaccination process experiences and perceptions among the 10,825 study participants

	n	%			n	%
COVID-19 experiences & perceptions				COVID-19 vaccination expectations & perceptions		
How fearful are you about getting COVID-19?				How important is it for you to receive COVID-19 vaccination?		
Very fearful	2773	25.6		Very important	2665	24.6
A little fearful	1685	15.6		Important	3265	30.2
Not sure	1914	17.7		Not sure	2346	21.7
Not fearful	1913	17.7		Not important	1564	14.4
Not fearful at all	2540	23.4		Not important at all	985	9.1
How possible is it for you to get COVID-19?				How fearful are you about having severe side-effects from COVID-19 vaccination?		
Highly possible	2291	21.2		Not fearful at all	2186	20.2
A bit possible	1407	13.0		Not fearful	2253	20.8
Not sure	2319	21.4		Not sure	2475	22.9
Not possible	1821	16.8		A little fearful	1653	15.3
Not possible at all	2987	27.6		Very fearful	2258	20.8
How possible is it for you to get severe COVID-19?				What protection against COVID-19 will the vaccination give?		
Highly possible	2154	19.9		Full protection	3925	36.3
A bit possible	1349	12.5		Partial protection	1655	15.3
Not sure	2241	20.7		Not sure	3573	33.0
Not possible	1780	16.4		No protection	880	8.1
Not possible at all	3301	30.5		No protection at all	792	7.3
Have you ever had COVID-19?				How do you trust the health workers giving the vaccination?		
Yes, surely	365	3.4		Trust them very much	2545	23.5
Yes, think so	195	1.8		Trust them	3739	34.5
Not sure	2386	22.0		Not sure	2478	22.9
No, think so	1233	11.4		Do not trust them	1239	11.5
No, surely	6646	61.4		Do not trust them at all	824	7.6
Have you ever had severe COVID-19?				How do you trust the government providing the vaccination?		
Yes, very serious	286	2.6		Trust them very much	2767	25.6
Yes, a bit serious	125	1.2		Trust them	3403	31.4
Not sure	111	1.0		Not sure	2437	22.5
No, not serious	7	0.1		Do not trust them	1296	12.0
No, not serious at all	31	0.3		Do not trust them at all	922	8.5
				**COVID-19 vaccination process experiences & perceptions**		
Know any person who have had COVID-19?				Ever heard COVID-19 vaccination was available for receipt?		
Yes, a very close person	343	3.2		Yes, many times	6175	57.0
Yes, a close person	225	2.1		Yes, once/few times	1747	16.2
Yes, a distant person	201	1.8		Not sure	686	6.3
Yes, a very distant person	262	2.4		No, no time	679	6.3
No	9794	90.5		No, no time at all	1538	14.2
Know any person who have had severe COVID-19?				Know a COVID-19 vaccination place?		
Yes, a very close person	265	2.5		Yes, a very close place	3249	30.0
Yes, a close person	162	1.5		Yes, a close place	1887	17.4
Yes, a distant person	198	1.8		Yes, a far place	765	7.1
Yes, a very distant person	207	1.9		Yes, a very far place	386	3.6
No	199	1.8		No	4538	41.9
Know any person who have died from COVID-19?				Frequency of COVID-19 vaccination at that place?		
Yes, a very close person	198	1.8		Daily, down to twice a week	3199	29.5
Yes, a close person	129	1.2		Once a week	630	5.8
Yes, a distant person	203	1.9		Once in two–four weeks	300	2.8
Yes, a very distant person	189	1.7		No fixed time	439	4.1
No	312	2.9		Do not know	1719	15.9
				Queue at the vaccination place?		
				No queue	2733	25.2
				Short queue	1008	9.3
				Do not know	2273	21.0
				Long queue	200	1.9
				Very long queue	73	0.7
				How caring are the health workers at the vaccination place?		
				Very caring	2160	19.9
				Caring	2044	18.9
				Not sure	1862	17.2
				Not caring	160	1.5
				Not caring at all	61	0.6

### Predictors of COVID-19 and COVID-19 vaccination and the vaccination process experiences and perceptions

Prevalence estimates and crude and adjusted prevalence differences and their respective 97.5% CI and *p*-values are reported for each dichotomous or polychotomous independent factor while crude and adjusted coefficients are reported for each continuous independent factor (Tables 3, 4 and 5). The crude and adjusted *p*-values of the overall effect of each polychotomous independent factor are also reported (Tables 3, 4 and 5).

The extent of COVID-19 experience and perception and the associations between it and sociodemographic and background factors are presented in Table 3. Among the 10,825 study participants, 3420 (31.6%) had strong COVID-19 experience and perception while 7405 (68.4%) had not strong COVID-19 experience and perception. The adjusted results show that the predictors of strong COVID-19 experience and perception were: good attitude towards COVID-19 and COVID-19 vaccination (adjusted prevalence difference (aPD) 30.4%, 97.5% CI 20.8–40.0, *p* < 0.0001); good knowledge about COVID-19 (aPD 15.0%, 8.9–21.2, *p* < 0.0001); being married (aPD 4.2%, 1.6–6.8, *p* = 0.0003); main occupation (adjusted *p* value of overall effect = 0.0006); and educational level (adjusted *p* value of overall effect = 0.0117).

The level of COVID-19 vaccination expectation and perception and the associations between it and sociodemographic and background factors are presented in Table 4. Among the 10,825 study participants, 7813 (72.2%) had good COVID-19 vaccination expectation and perception while 3012 (27.8%) had poor COVID-19 vaccination expectation and perception. The predictors of good COVID-19 vaccination expectation and perception were: good attitude towards COVID-19 and COVID-19 vaccination (aPD 29.9%, 23.7–36.1, *p* < 0.0001); good knowledge about COVID-19 (aPD 9.3%, 3.2–15.5, *p* = 0.0007); and main occupation (adjusted *p* value of overall effect = 0.0228).

**Table 3:** Association between sociodemographic and background factors and the extent of COVID-19 experience and perception among the 10,825 study participants

	Extent of COVID-19 experience & perception^		Crude results*		Adjusted results**	
	**Strong** **n (%)** **3420 (31.6)**	**Not strong** **n (%)** **7405 (68.4)**	cPD (97.5% CI) or cCoef (97.5% CI)	p value	aPD (97.5% CI) or aCoef (97.5% CI)	***p*** value
Gender						
Male	1509 (31.8)	3240 (68.2)	0	–	0	–
Female	1911 (31.5)	4165 (68.5)	-0.1% (-2.5–2.3)	0.9362	1.0% (-1.2–3.2)	0.2943
Age, years (coefficient)	–	–	-0.002% (-0.1–0.1)	0.9633	-0.1% (-0.3–0.04)	0.1066
Marital status						
Not married^1^	1406 (27.5)	3707 (72.5)	0	–	0	–
Married	2014 (35.3)	3698 (64.7)	5.6% (2.3–8.9)	0.0002	4.2% (1.6–6.8)	0.0003
Educational level				0.0002^$^		0.0117^$^
No formal education	354 (33.2)	711 (66.8)	0	–	0	–
Primary	628 (28.4)	1583 (71.6)	1.4% (-4.2–7.1)	0.5679	-5.1% (-9.8–(-0.5))	0.0139
Secondary	1671 (27.5)	4412 (72.5)	2.9% (-3.0–8.9)	0.2670	-6.3% (-12.8–0.1)	0.0284
Tertiary	767 (52.3)	699 (47.7)	18.7% (8.7–28.6)	< 0.0001	1.1% (-5.9–8.1)	0.7218
Main occupation				< 0.0001^$^		0.0006^$^
Self-employment^2^	1835 (31.1)	4072 (68.9)	0	–	0	–
Private paid work	238 (33.1)	482 (66.9)	1.7% (-3.1–6.6)	0.4240	-2.9% (-6.5–0.7)	0.0687
Government paid work	396 (62.3)	240 (37.7)	21.8% (13.4–30.2)	< 0.0001	10.5% (4.2–16.8)	0.0002
Others^3^	951 (26.7)	2611 (73.3)	-1.7% (-5.9–2.5)	0.3638	1.6% (-5.9–9.2)	0.6225
Residence						
Urban or semi-urban	617 (25.6)	1792 (74.4)	0	–	0	–
Rural	2803 (33.3)	5613 (66.7)	7.2% (-6.8–21.3)	0.2491	7.0% (-7.5–21.6)	0.2789
Usual monthly income, NGN				< 0.0001^$^		0.0264^$^
No income	706 (23.7)	2274 (76.3)	0	–	0	–
20,000 and less	1416 (31.5)	3084 (68.5)	4.1% (-0.6–8.8)	0.0494	3.6% (-4.3–11.6)	0.3032
More than 20,000	1298 (38.8)	2047 (61.2)	11.2% (6.4–16.1)	< 0.0001	6.9% (0.02–13.8)	0.0247
Main source of information about COVID-19				0.6773^$^		0.3038^$^
Internet, social media (whatsapp, facebook), & SMS	209 (25.7)	604 (74.3)	0	–	0	–
Traditional media (television, radio, prints)	1656 (27.5)	4361 (72.5)	0.9% (-4.7–6.6)	0.7099	3.9% (-2.9–10.7)	0.1977
Interpersonal^4^	1555 (38.9)	2440 (61.1)	-2.0% (-9.5–5.4)	0.5404	0.1% (-8.5–8.7)	0.9810
Most trusted source of information about COVID-19				0.9776^$^		0.3205^$^
Internet, social media (whatsapp, facebook), & SMS	180 (27.8)	468 (72.2)	0	–	0	–
Traditional media (television, radio, prints)	1580 (26.9)	4303 (73.1)	0.04% (-5.6–5.7)	0.9867	-3.7% (-10.7–3.4)	0.2413
Interpersonal^4^	1660 (38.7)	2634 (61.3)	-0.6% (-7.7–6.4)	0.8417	0.5% (-6.1–7.1)	0.8577
Level of knowledge about COVID-19^5^						
Poor	2822 (29.0)	6909 (71.0)	0	–	0	–
Good	598 (54.7)	496 (45.3)	22.5% (13.9–31.1)	< 0.0001	15.0% (8.9–21.2)	< 0.0001
Level of attitude towards COVID-19 and COVID-19 vaccination^6^						
Poor	791 (15.0)	4469 (85.0)	0	–	0	–
Good	2629 (47.2)	2936 (52.8)	33.1% (23.1–43.2)	< 0.0001	30.4% (20.8–40.0)	< 0.0001

cPD = Crude prevalence difference. aPD = Adjusted prevalence difference. cCoef = Crude coefficient. aCoef = Adjusted coefficient. ^COVID-19 experiences and perceptions score of ≥ 50% of the highest attainable score of 32 was strong experience and perception and < 50% was not strong experience and perception. *Adjusted for clustering. **Adjusted for clustering; Basic knowledge of COVID-19; Attitude towards COVID-19 & COVID-19 vaccination; Source of information about COVID-19 (Main source and Most trusted source of information about COVID-19); and Sociodemographic characteristics (Gender, Age, Marital status, Educational level, Occupation, Residence (rural vs. urban or semi-urban), and Monthly income). ^$^*p* value of overall effect. ^1^Separated or Divorced or Widowed or Never married (Single). ^2^Farmer or Trader or Other self-employments. ^3^Housewife or Student or Apprentice or Youth Corper or None. ^4^Relatives/friends, health workers, place of work, place of worship etc. ^5^Knowledge score of < 75% of the highest attainable score of 44 was poor knowledge and ≥ 75% was good knowledge ^6^Attitude score of < 75% of the highest attainable score of 80 was poor attitude and ≥ 75% was good attitude

The level of COVID-19 vaccination process experience and perception and the associations between it and sociodemographic and background factors are presented in Table 5. Among the 10,825 study participants, 5869 (54.2%) had positive COVID-19 vaccination process experience and perception while 4956 (45.8%) had negative COVID-19 vaccination process experience and perception. The predictors of positive COVID-19 vaccination process experience and perception were: good attitude towards COVID-19 and COVID-19 vaccination (aPD 11.5%, 5.0–17.9, *p* < 0.0001); good knowledge about COVID-19 (aPD 10.4%, 4.3–16.5, *p* < 0.0001); being married (aPD 5.8%, 3.0–8.5, *p* < 0.0001); educational level (adjusted *p* value of overall effect = 0.0109); and age as one year increase in age reduces the probability of having positive COVID-19 vaccination process experience and perception by 0.2% (adjusted coefficient (aCoef) -0.2%, 97.5% CI -0.4–(-0.1), *p* = 0.0021).

**Table 4:** Association between sociodemographic and background factors and the COVID-19 vaccination expectation and perception level among the 10,825 study participants

	COVID-19 vaccination expectation & perception level^		Crude results*		Adjusted results**	
	**Good** **n (%)** **7813 (72.2)**	**Poor** **n (%)** **3012 (27.8)**	cPD (97.5% CI) or cCoef (97.5% CI)	***p*** value	aPD (97.5% CI) or aCoef (97.5% CI)	***p*** value
Gender						
Male	3387 (71.3)	1362 (28.7)	0	–	0	–
Female	4426 (72.8)	1650 (27.2)	0.7% (-1.6–2.9)	0.4992	0.8% (-0.9–2.5)	0.2835
Age, years (coefficient)	–	–	-0.03% (-0.2–0.1)	0.5665	-0.03% (-0.2–0.1)	0.6957
Marital status						
Not married^1^	3599 (70.4)	1514 (29.6)	0	–	0	–
Married	4214 (73.8)	1498 (26.2)	2.5% (-0.6–5.6)	0.0682	1.7% (-1.5–4.9)	0.2342
Educational level				0.0007^$^		0.5501^$^
No formal education	714 (67.0)	351 (33.0)	0	–	0	–
Primary	1641 (74.2)	570 (25.8)	4.6% (-2.5–11.6)	0.1490	0.2% (-6.7–7.0)	0.9588
Secondary	4270 (70.2)	1813 (29.8)	6.0% (-2.0–14.0)	0.0917	-0.6% (-8.7–7.5)	0.8653
Tertiary	1188 (81.0)	278 (19.0)	14.4% (5.3–23.5)	0.0004	2.0% (-6.1–10.1)	0.5818
Main occupation				< 0.0001^$^		0.0228^$^
Private paid work	522 (72.5)	198 (27.5)	0	–	0	–
Self-employment^2^	4258 (72.1)	1649 (27.9)	-2.0% (-6.9–2.9)	0.3586	1.3% (-3.2–5.8)	0.5156
Government paid work	546 (85.8)	90 (14.2)	10.7% (4.6–16.9)	0.0001	6.2% (1.6–10.9)	0.0028
Others^3^	2447 (69.8)	1075 (30.2)	-2.2% (-5.9–1.5)	0.1902	2.8% (-3.1–8.7)	0.2890
Residence						
Urban or semi-urban	1683 (69.9)	726 (30.1)	0	–	0	–
Rural	6130 (72.8)	2286 (27.2)	2.8% (-10.5–16.0)	0.6394	2.3% (-11.7–16.3)	0.7138
Usual monthly income, NGN				0.0136^$^		0.4257^$^
No income	2005 (67.3)	975 (32.7)	0	–	0	–
20,000 and less	3344 (74.3)	1155 (25.7)	3.6% (-0.7–8.0)	0.0624	3.7% (-2.7–10.2)	0.1943
More than 20,000	2464 (73.7)	881 (26.3)	6.0% (1.3–10.7)	0.0044	3.9% (-3.6–11.3)	0.2452
Main source of information about COVID-19				0.9530^$^		0.6380^$^
Internet, social media (whatsapp, facebook), & SMS	584 (71.8)	229 (28.2)	0	–	0	–
Traditional media (television, radio, prints)	4153 (69.0)	1864 (31.0)	-0.9% (-7.6–5.7)	0.7600	-1.9% (-7.6–3.8)	0.4565
Interpersonal^4^	3076 (77.0)	919 (23.0)	-0.7% (-8.4–7.1)	0.8478	-2.7% (-9.2–3.7)	0.3441
Most trusted source of information about COVID-19				0.7346^$^		0.0837^$^
Internet, social media (whatsapp, facebook), & SMS	454 (70.1)	194 (29.9)	0	–	0	–
Traditional media (television, radio, prints)	4055 (68.9)	1828 (31.1)	1.1% (-5.8–7.9)	0.7247	2.8% (-2.8–8.4)	0.2620
Interpersonal^4^	3304 (76.9)	990 (23.1)	2.8% (-5.7–11.3)	0.4662	6.8% (-0.2–13.8)	0.0307
Level of knowledge about COVID-19^5^						
Poor	6816 (70.0)	2915 (30.0)	0	–	0	–
Good	997 (91.1)	97 (8.9)	17.2% (10.9–23.5)	< 0.0001	9.3% (3.2–15.5)	0.0007
Level of attitude towards COVID-19 and COVID-19 vaccination^6^						
Poor	2957 (56.2)	2303 (43.8)	0	–	0	–
Good	4856 (87.3)	709 (12.7)	30.7% (24.1–37.3)	< 0.0001	29.9% (23.7–36.1)	< 0.0001

cPD = Crude prevalence difference. aPD = Adjusted prevalence difference. cCoef = Crude coefficient. aCoef = Adjusted coefficient. ^COVID-19 vaccination expectations and perceptions score of ≥ 50% of the highest attainable score of 20 was good expectation and perception and < 50% was poor expectation and perception. *Adjusted for clustering. **Adjusted for clustering; Basic knowledge of COVID-19; Attitude towards COVID-19 & COVID-19 vaccination; Source of information about COVID-19 (Main source and Most trusted source of information about COVID-19); and Sociodemographic characteristics (Gender, Age, Marital status, Educational level, Occupation, Residence (rural vs. urban or semi-urban), and Monthly income). ^$^*p* value of overall effect. ^1^Separated or Divorced or Widowed or Never married (Single). ^2^Farmer or Trader or Other self-employments. ^3^Housewife or Student or Apprentice or Youth Corper or None. ^4^Relatives/friends, health workers, place of work, place of worship etc. ^5^Knowledge score of < 75% of the highest attainable score of 44 was poor knowledge and ≥ 75% was good knowledge. ^6^Attitude score of < 75% of the highest attainable score of 80 was poor attitude and ≥ 75% was good attitude

**Table 5:** Association between sociodemographic and background factors and the COVID-19 vaccination process experience and perception level among the 10,825 study participants

	COVID-19 vaccination process experience & perception level^		Crude results*		Adjusted results**	
	**Positive** **n (%)** **5869 (54.2)**	**Negative** **n (%)** **4956 (45.8)**	cPD (97.5% CI) or cCoef (97.5% CI)	***p*** value	aPD (97.5% CI) or aCoef (97.5% CI)	***p*** value
Gender						
Male	2604 (54.8)	2145 (45.2)	0	–	0	–
Female	3265 (53.7)	2811 (46.3)	-0.2% (-2.6–2.1)	0.8190	0.9% (-1.3–3.0)	0.3686
Age, years (coefficient)	–	–	-0.1% (-0.2–0.1)	0.2043	-0.2% (-0.4–(-0.1))	0.0021
Marital status						
Not married^1^	2609 (51.0)	2504 (49.0)	0	–	0	–
Married	3260 (57.1)	2452 (42.9)	6.3% (3.3–9.3)	< 0.0001	5.8% (3.0–8.5)	< 0.0001
Educational level				< 0.0001^$^		0.0109^$^
No formal education	541 (50.8)	524 (49.2)	-7.1% (-13.6–(-0.5))	0.0161	1.3% (-6.4–9.1)	0.6995
Primary	1099 (49.7)	1112 (50.3)	-1.2% (-3.9–1.5)	0.3163	0.6% (-2.8–4.1)	0.6764
Secondary	3055 (50.2)	3028 (49.8)	0	–	0	–
Tertiary	1174 (80.1)	292 (19.9)	14.8% (8.3–21.2)	< 0.0001	8.4% (2.7–14.0)	0.0009
Main occupation				< 0.0001^$^		0.1166^$^
Self-employment^2^	3078 (52.1)	2829 (47.9)	0	–	0	–
Private paid work	469 (65.1)	251 (34.9)	6.1% (-0.4–12.7)	0.0367	0.7% (-0.56–7.1)	0.7964
Government paid work	539 (84.7)	97 (15.3)	18.0% (12.0–24.0)	< 0.0001	5.3% (-0.01–10.5)	0.0254
Others^3^	1783 (50.1)	1779 (49.9)	-2.3% (-5.9–1.4)	0.1633	2.1% (-3.7–7.8)	0.4246
Residence						
Urban or semi-urban	1398 (58.0)	1011 (42.0)	0	–	0	–
Rural	4471 (53.1)	3945 (46.9)	-4.9% (-32.4–22.6)	0.6884	-3.6% (-29.9–22.8)	0.7617
Usual monthly income, NGN				< 0.0001^$^		0.0257^$^
No income	1377 (46.2)	1603 (53.8)	0	–	0	–
20,000 and less	2326 (51.7)	2174 (48.3)	4.8% (1.0–8.5)	0.0044	5.4% (-0.6–11.3)	0.0436
More than 20,000	2166 (64.8)	1179 (35.2)	13.3% (6.6–20.0)	< 0.0001	11.2% (1.9–20.5)	0.0068
Main source of information about COVID-19				0.3329^$^		0.7901^$^
Internet, social media (whatsapp, facebook), & SMS	357 (43.9)	456 (56.1)	0	–	0	–
Traditional media (television, radio, prints)	2976 (49.46)	3041 (50.5)	1.7% (-4.2–7.6)	0.5218	-1.5% (-7.6–4.7)	0.5945
Interpersonal^4^	2536 (63.5)	1459 (36.5)	3.1% (-1.7–7.8)	0.1451	0.3% (-6.1–6.7)	0.9156
Most trusted source of information about COVID-19				0.1024^$^		0.1039^$^
Internet, social media (whatsapp, facebook), & SMS	301 (46.5)	347 (53.5)	0	–	0	–
Traditional media (television, radio, prints)	3008 (51.1)	2875 (48.9)	3.7% (-2.7–10.1)	0.1931	4.8% (-1.9–11.6)	0.1102
Interpersonal^4^	2560 (59.6)	1734 (40.4)	5.3% (-0.5–11.1)	0.0396	7.1% (-0.6–14.7)	0.0380
Level of knowledge about COVID-19^5^						
Poor	4961 (51.0)	4770 (49.0)	0	–	0	–
Good	908 (83.0)	186 (17.0)	16.8% (9.0–24.6)	< 0.0001	10.4% (4.3–16.5)	0.0001
Level of attitude towards COVID-19 and COVID-19 vaccination^6^						
Poor	2421 (46.0)	2839 (54.0)	0	–	0	–
Good	3448 (62.0)	2117 (38.0)	13.9% (7.5–20.4)	< 0.0001	11.5% (5.0–17.9)	0.0001

cPD = Crude prevalence difference. aPD = Adjusted prevalence difference. cCoef = Crude coefficient. aCoef = Adjusted coefficient. ^COVID-19 vaccination process experiences and perceptions score of ≥ 50% of the highest attainable score of 20 was positive experience and perception and < 50% was negative experience and perception. *Adjusted for clustering. **Adjusted for clustering; Basic knowledge of COVID-19; Attitude towards COVID-19 & COVID-19 vaccination; Source of information about COVID-19 (Main source and Most trusted source of information about COVID-19); and Sociodemographic characteristics (Gender, Age, Marital status, Educational level, Occupation, Residence (rural vs. urban or semi-urban), and Monthly income). ^$^*p* value of overall effect. ^1^Separated or Divorced or Widowed or Never married (Single). ^2^Farmer or Trader or Other self-employments. ^3^Housewife or Student or Apprentice or Youth Corper or None. ^4^Relatives/friends, health workers, place of work, place of worship etc. ^5^Knowledge score of < 75% of the highest attainable score of 44 was poor knowledge and ≥ 75% was good knowledge.^6^Attitude score of < 75% of the highest attainable score of 80 was poor attitude and ≥ 75% was good attitude

### Predictors of dichotomized (positive and non-positive) COVID-19 and COVID-19 vaccination and the vaccination process experiences and perceptions

These results are presented in the appendix. Regarding COVID-19 experiences and perceptions: 4458 (41.2%) were fearful of getting COVID-19 while 6367 (58.8%) were not fearful/not sure and the predictors of being fearful of getting COVID-19 were good attitude towards COVID-19 and COVID-19 vaccination and decrease in age (appendix p 2). 3698 (34.2%) said it was possible for them to get COVID-19 while 7127 (65.8%) said it was not possible or that they were not sure about it and the predictors of having the perception that it was possible to get COVID-19 were good attitude towards COVID-19 and COVID-19 vaccination, good knowledge about COVID-19, and main occupation (appendix p 4).

Regarding COVID-19 vaccination expectations and perceptions, 5930 (54.8%) said it was important for them to receive COVID-19 vaccination while 4895 (45.2%) said it was not important or that they were not sure about it. The predictors of having the perception that it was important to receive COVID-19 vaccination were good attitude towards COVID-19 and COVID-19 vaccination, good knowledge about COVID-19, female gender, main occupation, and level of monthly income (appendix p 6). 4439 (41.0%) were not fearful of having severe side-effects from COVID-19 vaccination while 6386 (59.0%) were fearful or not sure about it. The predictors of not being fearful of having severe side-effects from COVID-19 vaccination were good attitude towards COVID-19 and COVID-19 vaccination, good knowledge about COVID-19, and level of monthly income (appendix p 8). 5580 (51.6%) said COVID-19 vaccination would give them protection against COVID-19 while 5245 (48.4%) said it would give no protection or that they were not sure about it. The predictors of having the perception that COVID-19 vaccination would give protection against COVID-19 were good attitude towards COVID-19 and COVID-19 vaccination, being married, educational, and decrease in age (appendix p 10).

Regarding COVID-19 vaccination process experiences and perceptions, 7922 (73.2%) had heard COVID-19 vaccination was available for them to go and receive while 2903 (26.8%) had not heard or were not sure about it. The predictors of being aware COVID-19 vaccination was available for receipt were good attitude towards COVID-19 and COVID-19 vaccination, being married, and educational level (appendix p 12). 5136 (47.5%) knew a close COVID-19 vaccination place while 5689 (52.5%) knew a far place or no place and the predictors of knowing a close COVID-19 vaccination place were good attitude towards COVID-19 and COVID-19 vaccination, being married, and decrease in age (appendix p 14).

## Discussion

This study assessed and explored the experiences and perceptions about COVID-19 and COVID-19 vaccination and its process, and their predictors, among community members during the COVID-19 pandemic in Ebonyi state, Nigeria. According to the study findings: 31.6% had strong COVID-19 experience and perception and the predictors were good attitude towards COVID-19 and COVID-19 vaccination, good knowledge about COVID-19, being married, main occupation, and educational level; 72.2% had good COVID-19 vaccination expectation and perception and the predictors were good attitude towards COVID-19 and COVID-19 vaccination, good knowledge about COVID-19, and main occupation; and 54.2% had positive COVID-19 vaccination process experience and perception and the predictors were good attitude towards COVID-19 and COVID-19 vaccination, good knowledge about COVID-19, being married, educational level, and decrease in age.

Considering the uniqueness of the above outcome measures explored by the study, we did not identify any relevant studies with comparable outcomes for appropriate comparison of findings. However, the low prevalence (31.6%) of strong COVID-19 experience and perception was consistent with the fact that the pandemic was relatively less severe in Ebonyi state and in Nigeria as fewer COVID-19 cases and related deaths were confirmed compared to many other countries.

The relatively high prevalence (72.2%) of good COVID-19 vaccination expectation and perception despite the misinformation/disinformation and conspiracy theories about COVID-19 and COVID-19 vaccination in the media indicate that this misinformation/disinformation had limited negative effects on people’s perceptions about COVID-19 vaccination. A plausible explanation could be that, because this study was conducted in the prevailing context of increased availability and access to actual vaccines, the real experiences and close observations and perceptions of the importance, safety/side-effects, and effectiveness of the vaccination among those who were, or knew others who were, already vaccinated, could have influenced their perceptions about the vaccination despite the misinformation/disinformation. However, further studies, especially qualitative studies, are required to provide more insights. The above explanation implies that the negative effects of COVID-19 and COVID-19 vaccination misinformation/disinformation on people’s perceptions about COVID-19 vaccination decreased over the course of the pandemic. This is consistent with the reported evidence that people’s trust in COVID-19 information on the social media declined over time during the pandemic [10]. This was an important finding because the social media was perhaps the foremost channel for most COVID-19 misinformation/disinformation before such information were further circulated via other traditional/interpersonal channels. Another possible factor that could have contributed to the limited negative effects of misinformation/disinformation was the fact that while the misinformation/disinformation was more in the social media, traditional media such as the radio was the main and most trusted source of information for majority of the community members in the study area (as we have observed during the study).

The prevalence of 54.2% of positive COVID-19 vaccination process experience and perception indicate that in the prevailing context of increased availability and access to COVID-19 vaccination in Ebonyi state, Nigeria, more needed to be done by the government and health care leaders to improve the ease of access and the appeal of the COVID-19 vaccination system. We did not identify relevant studies for comparison, however, the predictors identified by this study highlight factors that should be considered by policy makers/health leaders in the design and implementation of strategies to improve COVID-19 and COVID-19 vaccination and the vaccination process experiences and perceptions in Ebonyi state, Nigeria and other similar settings.

In our study, 41.2% were fearful of getting COVID-19, 34.2% said it was possible for them to get COVID-19, 41.0% were not fearful of having severe side-effects from COVID-19 vaccination, and 51.6% said COVID-19 vaccination would give them protection against COVID-19. A relatively higher proportion of 50.7% were fearful of getting COVID-19 in a study in Nigeria, [11] however, this study was conducted much earlier during the initial waves of the pandemic (in the later-half of 2020) when there was much uncertainty, confusion, and fear/anxiety and this timing could have accounted for the higher value. Moreover, this other study was only online (unlike our study that was a geographical community-based offline survey among participants in both rural and urban/semi-urban areas) and difference in socioeconomic status between the participants of both studies could have partly accounted for the contrasting findings. Another study in Nigeria reported only 26.0% who perceived they were at risk of getting COVID-19 [12]. This lower value could have resulted from the fact that this study had a relatively lower sample size of 360 across only three LGAs in a state and was conducted in mid-2020 when the awareness of and cases of COVID-19 were much lower in Nigeria. Respectively higher proportions of 56.4% perceived they were at risk (low risk or medium to very high risk) of getting COVID-19, 60.9% were confident that the vaccination was safe, and 59.9% were confident that the vaccination was effective in a study in Tanzania [13]. Similarly, higher proportions of 47.5% in a study in the UK,[14] 56.3% in Malaysia,^6^ 59.6% in Iran,[15] and 89.0% in Hong Kong [16] perceived it was likely for them to get COVID-19. However, a lower proportion of 33.7% in Lebanon [17] were afraid of COVID-19.

Our study identified good attitude towards COVID-19 and COVID-19 vaccination and decrease in age as predictors of being fearful of getting COVID-19 and good attitude towards COVID-19 and COVID-19 vaccination, good knowledge about COVID-19, and main occupation as predictors of having the perception that it was possible to get COVID-19. In comparison, the predictors of fear of COVID-19 in other relevant studies include male in a study in Nigeria, [11] and marital status (married/divorced versus single) in Lebanon [17]. It is worth noting that we did not find gender to be a predictor of being fearful of getting COVID-19 or of having the perception that it was possible to get COVID-19 and this was consistent with findings of studies in Lebanon [17], However, other studies have reported that female gender was a predictor of COVID-19 related fear [18–20] and of perceived susceptibility to COVID-19, [20] although, there were great variations regarding this evidence between studies across different continents as the effect of gender on COVID-19 related fear was more pronounced in European studies [18]. 

The predictors of having confidence in COVID-19 vaccination (not being fearful of severe side-effects and perceiving the vaccination is protective) identified by our study include good attitude towards COVID-19 and COVID-19 vaccination, good knowledge about COVID-19, level of monthly income, being married, educational level, and decrease in age. In comparison, the predictors of having confidence in COVID-19 vaccine were good knowledge of COVID-19 vaccine and rural residence in Tanzania [13]. 

The reasons for some of the above contrasting findings are not clear but could perhaps be due to contextual differences between the study settings. For example, he lower level of perceived susceptibility/possibility of getting infected with COVID − 19 in our study compared to the UK and Hong Kong studies might be as a result of the lower severity of the pandemic in Ebonyi state/Nigeria compared to these countries because the severity of the pandemic influenced people’s COVID-19 risk perception [21]. Another reason could be time trends in the perceptions of COVID-19 and COVID-19 vaccination as most of these studies were conducted much earlier than our study and during the initial waves of the pandemic when there was much uncertainty, confusion, fear, and anxiety and when there were no real experiences of the importance, safety/side-effects, and effectiveness of actual COVID-19 vaccines/vaccination. Also, most of these other studies were conducted online (unlike our study that was a geographical community-based offline survey among participants in both rural and urban/semi-urban areas) and difference in socioeconomic status between the participants of our study and these other studies could have partly explained the contrasting findings.

In our study, 73.2% had heard COVID-19 vaccination was available for them to go and receive. In comparison, a higher proportion of 93.8% in a study in Tanzania [22] had heard about COVID-19 vaccines. The lower value in our study was perhaps due to specific nature of our outcome (the awareness that COVID-19 vaccination was available for them to go and receive) which particularly reflect the receipt of the health message that people should go and receive COVID-19 vaccination to protect against COVID-19. In our study, 58.1% knew a COVID-19 vaccination place (the combination of those who knew a very close place, a close place, a far place, and a very far place) and this value is not too different from the 63.9% who knew where to get COVID-19 vaccination in Tanzania [22]. The slight difference could be due to contextual factors.

A limitation in this study was reporting bias which is associated with questionnaire-based studies. The outcome measurement involved participants reporting their COVID-19 and COVID-19 vaccination and the vaccination process experiences and perceptions and, as a result, there could be recall bias because some of these experiences and perceptions were past events. But the bias would be minimal because such experiences and perceptions were largely ongoing. Also, the outcomes were related to COVID-19/COVID-19 vaccination which was a controversial topic due to the misinformation/disinformation and conspiracy theories and, as a result, there was the tendency for some respondents to exaggerate good/desirable perceptions and underestimate perceptions that were not good/desirable. However, this bias would be minimal because the anonymous and confidential nature of the questionnaire survey were duly explained and emphasized to the respondents.

This study had some strengths. It was a geographical-community based study (not online based) that involved participants in both rural and urban/semi-urban areas of Ebonyi state, Nigeria. Thus, the study findings are more generalisable to the entire state and other states in Nigeria, including other poor resource settings with limited internet access. Also, the outcome measures and the potential covariates were pre-specified as the study was registered prospectively and implemented based on a study protocol which was prospectively submitted to a peer-review journal.

## Conclusions

There was low prevalence of strong COVID-19 experience and perception, high prevalence of good COVID-19 vaccination expectation and perception, and moderate level of prevalence of positive COVID-19 vaccination process experience and perception among the community members during the COVID-19 pandemic in Ebonyi state, Nigeria. The most important predictors of the extent/level of COVID-19 and COVID-19 vaccination and the vaccination process experiences and perceptions were level of attitude towards COVID-19 and COVID-19 vaccination and level of knowledge about COVID-19. Other important predictors were marital status, educational level, and main occupation. This evidence should inform subsequent relevant policy actions and interventions, including behaviour change communication strategies, regarding COVID-19 and COVID-19 vaccination and similar diseases in Ebonyi state, Nigeria, and other similar contexts. Further studies, preferably qualitative, are needed on the factors that influence COVID-19 and COVID-19 vaccination and the vaccination process experiences and perceptions and particularly on the (positive) effects of people’s real experiences and close perceptions of COVID-19 vaccination attributes (importance, safety/side-effects, effectiveness) on COVID-19 vaccination expectations and perceptions amidst (the negative effects of) misinformation/disinformation, and conspiracy theories.

### Electronic supplementary material

Below is the link to the electronic supplementary material.

Supplementary Material 1

## Data Availability

The datasets used and/or analysed during the current study are available from the corresponding author on reasonable request.
