# Supplementary material for: COVID-19 and COVID-19 vaccination experiences and perceptions and their predictors among community members during the COVID-19 pandemic in Ebonyi state, Nigeria: an analytical cross-sectional study
Source: BMC Public Health. 2024 Feb 22;24:567. doi: 10.1186/s12889-024-18028-5 (PMC10885597; doi:10.1186/s12889-024-18028-5)
Supplement: Supplementary file 1 — Supplementary Material 1 [file 12889_2024_18028_MOESM1_ESM.docx]

**APPENDIX**

COVID-19 and COVID-19 vaccination (process) experiences and perceptions and their predictors among community members during the COVID-19 pandemic in Ebonyi state, Nigeria: an analytical cross-sectional study

The results for the dichotomized (positive and non-positive) COVID-19 and COVID-19 vaccination (process) experiences and perceptions and their predictors are presented in this appendix.

**Table of Content**

Table 1: Association between sociodemographic and background factors and the fear of getting COVID-19 2

Table 2: Association between sociodemographic and background factors and
the perceived possibility of getting COVID-19 4

Table 3: Association between sociodemographic and background factors and
the perceived importance of receiving COVID-19 vaccination 6

Table 4: Association between sociodemographic and background factors and
the fear of having severe side-effects from COVID-19 vaccination 8

Table 5: Association between sociodemographic and background factors and
the perceived protection against COVID-19 from receiving COVID-19 vaccination 10

Table 6: Association between sociodemographic and background factors and
the awareness of the availability of COVID-19 vaccination for receipt 12

Table 7: Association between sociodemographic and background factors and
the knowledge of COVID-19 vaccination place 14

| Table 1: Association between sociodemographic and background factors and the fear of getting COVID-19 among the 10825 study participants | | | | | | |
| --- | --- | --- | --- | --- | --- | --- |
|  | **Fear of getting COVID-19** | | **Crude results*** | | **Adjusted results**** | |
|  | **Fearful^**  **n (%)**  **4458 (41.2)** | **Not fearful/not sure^^**  **n (%)**  **6367 (58.8)** | cPD (97.5% CI) or  cCoef (97.5% CI) | p value | aPD (97.5% CI) or  aCoef (97.5% CI) | p value |
| Gender |  |  |  |  |  |  |
| Male | 1921 (40.5) | 2828 (59.5) | 0 | – | 0 | – |
| Female | 2537 (41.8) | 3539 (58.2) | 1.6% (-0.7–3.9) | 0.1108 | 1.8% (-0.9–4.5) | 0.1293 |
| Age, years (coefficient) | – | – | -0.1% (-0.3–0.02) | 0.0475 | -0.2% (-0.4–(-0.02)) | 0.0117 |
| Marital status |  |  |  |  |  |  |
| Not married^1^ | 2095 (41.0) | 3018 (59.0) | 0 | – | 0 | – |
| Married | 2363 (41.4) | 3349 (58.6) | 1.2% (-2.3–4.7) | 0.4361 | 1.8% (-1.3–4.9) | 0.1971 |
| Educational level |  |  |  | 0.0705^$^ |  | 0.0351^$^ |
| No formal education | 413 (38.8) | 652 (61.2) | 0 | – | 0 | – |
| Primary | 766 (34.6) | 1445 (65.4) | -2.0% (-8.0–3.9) | 0.4424 | -7.7% (-13.8–(-1.6)) | 0.0047 |
| Secondary | 2477 (40.7) | 3606 (59.3) | 2.8% (-4.6–10.1) | 0.3966 | -6.3% (-12.7–0.2) | 0.0294 |
| Tertiary | 802 (54.7) | 664 (45.3) | 8.8% (-2.3–20.0) | 0.0760 | -4.4% (-12.9–4.1) | 0.2456 |
| Main occupation |  |  |  | 0.0438^$^ |  | 0.3690^$^ |
| Self-employment^2^ | 2327 (39.4) | 3580 (60.6) | 0 | – | 0 | – |
| Private paid work | 336 (46.7) | 384 (53.3) | 3.1% (-3.1–9.3) | 0.2602 | -0.4% (-5.1–4.4) | 0.8631 |
| Government paid work | 358 (56.3) | 278 (43.7) | 9.9% (1.6–18.1) | 0.0075 | 4.2% (-1.1–9.6) | 0.0777 |
| Others^3^ | 1437 (40.3) | 2125 (59.7) | 0.5% (-3.5–4.5) | 0.7717 | 1.0% (-6.5–8.4) | 0.7640 |
| Residence |  |  |  |  |  |  |
| Rural | 3270 (38.8) | 5146 (61.2) | 0 | – | 0 | – |
| Urban or semi-urban | 1188 (49.3) | 1221 (50.7) | 8.5% (-5.2–22.3) | 0.1648 | 8.1% (-5.7–21.9) | 0.1868 |
| Usual monthly income, NGN |  |  |  | 0.0458^$^ |  | 0.0840^$^ |
| No income | 1050 (35.2) | 1930 (64.8) | 0 | – | 0 | – |
| 20,000 and less | 1786 (39.7) | 2714 (60.3) | 2.1% (-3.1–7.3) | 0.3685 | 3.7% (-5.1–12.4) | 0.3492 |
| More than 20,000 | 1622 (48.5) | 1723 (51.5) | 5.9% (0.4–11.5) | 0.0155 | 7.0% (-2.6–16.6) | 0.1002 |
| Main source of information about COVID-19 |  |  |  | 0.8457^$^ |  | 0.9639^$^ |
| Internet, social media (whatsapp, facebook), & SMS | 306 (37.6) | 507 (62.4) | 0 | – | 0 | – |
| Traditional media (television, radio, prints) | 2312 (38.4) | 3705 (61.6) | -1.5% (-7.9–4.8) | 0.5949 | 0.8% (-5.6–7.1) | 0.7866 |
| Interpersonal^4^ | 1840 (46.1) | 2155 (53.9) | -1.5% (-9.0–6.0) | 0.6526 | 0.4% (-6.7–7.5) | 0.9045 |
| Most trusted source of information about COVID-19 |  |  |  | 0.8972^$^ |  | 0.8067^$^ |
| Internet, social media (whatsapp, facebook), & SMS | 238 (36.7) | 410 (63.3) | 0 | – | 0 | – |
| Traditional media (television, radio, prints) | 2273 (38.6) | 3610 (61.4) | -1.3% (-8.0–5.4) | 0.6673 | -1.0% (-9.6–7.5) | 0.7897 |
| Interpersonal^4^ | 1947 (45.3) | 2347 (54.7) | -0.3% (-10.3–9.7) | 0.9498 | 1.3% (-13.1–15.7) | 0.8369 |
| Level of knowledge about COVID-19^5^ |  |  |  |  |  |  |
| Poor | 3878 (39.9) | 5853 (60.1) | 0 | – | 0 | – |
| Good | 580 (53.0) | 514 (47.0) | 10.2% (-3.6–24.0) | 0.0987 | 5.2% (-6.2–16.6) | 0.3031 |
| Level of attitude towards COVID-19 (vaccination)^6^ |  |  |  |  |  |  |
| Poor | 1527 (29.0) | 3733 (71.0) | 0 | – | 0 | – |
| Good | 2931 (52.7) | 2634 (47.3) | 20.7% (9.2–32.3) | 0.0001 | 19.8% (8.8–30.9) | 0.0001 |
| ^Very fearful or a little fearful. ^^Not fearful at all, not fearful, or not sure. cPD=Crude prevalence difference. aPD=Adjusted prevalence difference. cCoef=Crude coefficient. aCoef=Adjusted coefficient. *Adjusted for clustering. **Adjusted for clustering; Basic knowledge of COVID-19; Attitude towards COVID-19 & COVID-19 vaccination; Source of information about COVID-19 (Main source and Most trusted source of information about COVID-19); and Sociodemographic characteristics (Gender, Age, Marital status, Educational level, Occupation, Residence (rural vs urban or semi-urban), and Monthly income). ^$^p value of overall effect. ^1^Separated or Divorced or Widowed or Never married (Single). ^2^Farmer or Trader or Other self-employments. ^3^Housewife or Student or Apprentice or Youth Corper or None. ^4^Relatives/friends, health workers, place of work, place of worship etc. ^5^Knowledge score of <75% of the highest attainable score of 44 was poor knowledge and >=75% was good knowledge. ^6^Attitude score of <75% of the highest attainable score of 80 was poor attitude and >=75% was good attitude. | | | | | | |

The fear of getting COVID-19 and the associations between it and sociodemographic and background factors are presented in table 1. Among the 10825 study participants, 4458 (41.2%) were fearful of getting COVID-19 while 6367 (58.8%) were not fearful/not sure. The adjusted results show that the predictors of being fearful of getting COVID-19 were good attitude towards COVID-19 (vaccination) (adjusted prevalence difference (aPD) 19.8%, 97.5% CI 8.8–30.9, p=0.0001) and age as one year increase in age reduces the probability of being fearful of getting COVID-19 by 0.2% (adjusted coefficient (aCoef) -0.2%, 97.5% CI -0.4–(-0.02), p=0.0117).

| Table 2: Association between sociodemographic and background factors and the perceived possibility of getting COVID-19 among the 10825 study participants | | | | | | |
| --- | --- | --- | --- | --- | --- | --- |
|  | **Possibility of getting COVID-19** | | **Crude results*** | | **Adjusted results**** | |
|  | **Possible^**  **n (%)**  **3698 (34.2)** | **Not possible/not sure^^**  **7127 (65.8)** | cPD (97.5% CI) or  cCoef (97.5% CI) | p value | aPD (97.5% CI) or  aCoef (97.5% CI) | p value |
| Gender |  |  |  |  |  |  |
| Male | 1607 (33.8) | 3142 (66.2) | 0 | – | 0 | – |
| Female | 2091 (34.4) | 3985 (65.6) | 1.1% (-1.7–3.9) | 0.3720 | 2.4% (-0.3–5.1) | 0.0457 |
| Age, years (coefficient) | – | – | -0.1% (-0.2–0.1) | 0.4428 | -0.1% (-0.3–0.1) | 0.2158 |
| Marital status |  |  |  |  |  |  |
| Not married^1^ | 1654 (32.4) | 3459 (67.6) | 0 | – | 0 | – |
| Married | 2044 (35.8) | 3668 (64.2) | 4.2% (1.0–7.4) | 0.0032 | 2.9% (-0.8–6.7) | 0.0821 |
| Educational level |  |  |  | 0.0013^$^ |  | 0.5769^$^ |
| No formal education | 224 (21.0) | 841 (79.0) | 0 | – | 0 | – |
| Primary | 747 (33.8) | 1464 (66.2) | 6.1% (-3.9–16.2) | 0.1709 | -0.8% (-9.6–8.0) | 0.8419 |
| Secondary | 1962 (32.3) | 4121 (67.7) | 7.9% (1.7–14.0) | 0.0044 | -1.4% (-8.4–5.6) | 0.6529 |
| Tertiary | 765 (52.2) | 701 (47.8) | 18.6% (7.7–29.4) | 0.0001 | 2.1% (-6.4–10.6) | 0.5825 |
| Main occupation |  |  |  | <0.0001^$^ |  | 0.0010^$^ |
| Self-employment^2^ | 1928 (32.6) | 3979 (67.4) | 0 | – | 0 | – |
| Private paid work | 359 (41.4) | 422 (58.6) | 3.6% (-2.2–9.4) | 0.1640 | -0.4% (-5.3–4.5) | 0.8688 |
| Government paid work | 359 (56.5) | 277 (43.5) | 17.3% (9.1–25.6) | <0.0001 | 8.8% (3.5–14.1) | 0.0002 |
| Others^3^ | 1113 (31.3) | 2449 (68.7) | -1.3% (-5.2–2.5) | 0.4310 | 1.1% (-3.7–5.8) | 0.6208 |
| Residence |  |  |  |  |  |  |
| Rural | 2669 (31.7) | 5747 (68.3) | 0 | – | 0 | – |
| Urban or semi-urban | 1029 (42.7) | 1380 (57.3) | 7.9% (-12.9–28.6) | 0.3955 | 8.4% (-12.5–29.2) | 0.3686 |
|  |  |  |  |  |  |  |
| Usual monthly income, NGN |  |  |  | <0.0001^$^ |  | 0.0471^$^ |
| No income | 698 (23.4) | 2282 (76.6) | 0 | – | 0 | – |
| 20,000 and less | 1572 (34.9) | 2928 (65.1) | 5.2% (-0.5–10.8) | 0.0399 | 4.7% (-1.0–10.5) | 0.0656 |
| More than 20,000 | 1428 (42.7) | 1917 (57.3) | 9.7% (5.2–14.2) | <0.0001 | 6.0% (0.4–11.7) | 0.0159 |
| Main source of information about COVID-19 |  |  |  | 0.5147^$^ |  | 0.9203^$^ |
| Internet, social media (whatsapp, facebook), & SMS | 238 (29.3) | 575 (70.7) | 0 | – | 0 | – |
| Traditional media (television, radio, prints) | 1872 (31.1) | 4145 (68.9) | 1.2% (-7.7–10.1) | 0.7685 | -0.9% (-12.3–10.6) | 0.8638 |
| Interpersonal^4^ | 1588 (39.8) | 2407 (60.2) | -3.2% (-14.3–7.9) | 0.5166 | 0.2% (-12.9–13.2) | 0.9774 |
| Most trusted source of information about COVID-19 |  |  |  | 0.2944^$^ |  | 0.2629^$^ |
| Internet, social media (whatsapp, facebook), & SMS | 167 (25.8) | 481 (74.2) | 0 | – | 0 | – |
| Traditional media (television, radio, prints) | 1959 (33.3) | 3924 (66.7) | 3.6% (-7.1–14.2) | 0.4536 | 3.8% (-10.4–18.0) | 0.5524 |
| Interpersonal^4^ | 1572 (36.6) | 2722 (63.4) | -2.6% (-14.0–8.8) | 0.6122 | -1.2% (-19.4–17.0) | 0.8828 |
| Level of knowledge about COVID-19^5^ |  |  |  |  |  |  |
| Poor | 2953 (30.4) | 6778 (69.6) | 0 | – | 0 | – |
| Good | 745 (68.1) | 349 (31.9) | 25.3% (10.4–40.3) | 0.0001 | 18.1% (3.8–32.4) | 0.0047 |
| Level of attitude towards COVID-19 (vaccination)^6^ |  |  |  |  |  |  |
| Poor | 872 (16.6) | 4388 (83.4) | 0 | – | 0 | – |
| Good | 2826 (50.8) | 2739 (49.2) | 29.2% (16.9–41.4) | <0.0001 | 26.6% (14.7–38.6) | <0.0001 |
| ^Highly possible or a bit possible. ^^Not possible at all, not possible, or not sure. cPD=Crude prevalence difference. aPD=Adjusted prevalence difference. cCoef=Crude coefficient. aCoef=Adjusted coefficient. *Adjusted for clustering. **Adjusted for clustering; Basic knowledge of COVID-19; Attitude towards COVID-19 & COVID-19 vaccination; Source of information about COVID-19 (Main source and Most trusted source of information about COVID-19); and Sociodemographic characteristics (Gender, Age, Marital status, Educational level, Occupation, Residence (rural vs urban or semi-urban), and Monthly income). ^$^p value of overall effect. ^1^Separated or Divorced or Widowed or Never married (Single). ^2^Farmer or Trader or Other self-employments. ^3^Housewife or Student or Apprentice or Youth Corper or None. ^4^Relatives/friends, health workers, place of work, place of worship etc. ^5^Knowledge score of <75% of the highest attainable score of 44 was poor knowledge and >=75% was good knowledge. ^6^Attitude score of <75% of the highest attainable score of 80 was poor attitude and >=75% was good attitude. | | | | | | |

The perceived possibility of getting COVID-19 and the associations between it and sociodemographic and background factors are presented in table 2. Among the 10825 study participants, 3698 (34.2%) said it was possible for them to get COVID-19 while 7127 (65.8%) said it was not possible or that they were not sure about it. The predictors of having the perception that it was possible to get COVID-19 were: good attitude towards COVID-19 (vaccination) (aPD 26.6%, 14.7–38.6, p<0.0001); good knowledge about COVID-19 (aPD 18.1%, 3.8–32.4, p=0.0047); and main occupation (adjusted p value of overall effect=0.0010).

| Table 3: Association between sociodemographic and background factors and the perceived importance of receiving COVID-19 vaccination among the 10825 study participants | | | | | | |
| --- | --- | --- | --- | --- | --- | --- |
|  | **Important to receive COVID-19 vaccination** | | **Crude results*** | | **Adjusted results**** | |
|  | **Important^**  **n (%)**  **5930 (54.8)** | **Not important/ not sure^^**  **n (%)**  **4895 (45.2)** | cPD (97.5% CI) or  cCoef (97.5% CI) | p value | aPD (97.5% CI) or  aCoef (97.5% CI) | p value |
| Gender |  |  |  |  |  |  |
| Male | 2516 (53.0) | 2233 (47.0) | 0 | – | 0 | – |
| Female | 3414 (56.2) | 2662 (43.8) | 1.8% (-1.2–4.7) | 0.1835 | 2.4% (0.2–4.6) | 0.0129 |
| Age, years (coefficient) | – | – | -0.1% (-0.3–0.05) | 0.1212 | -0.2% (-0.3–0.03) | 0.0544 |
| Marital status |  |  |  |  |  |  |
| Not married^1^ | 2654 (51.9) | 2459 (48.1) | 0 | – | 0 | – |
| Married | 3276 (57.4) | 2436 (42.6) | 3.5% (-0.6–7.5) | 0.0533 | 3.1% (-0.8–7.0) | 0.0715 |
| Educational level |  |  |  | <0.0001^$^ |  | 0.0481^$^ |
| No formal education | 525 (49.3) | 540 (50.7) | 0 | – | 0 | – |
| Primary | 1178 (53.3) | 1033 (46.7) | 3.3% (-3.7–10.4) | 0.2906 | -5.0% (-10.7–0.8) | 0.0523 |
| Secondary | 3173 (52.2) | 2910 (47.8) | 7.6% (0.06–15.2) | 0.0239 | -4.9% (-11.6–1.8) | 0.1028 |
| Tertiary | 1054 (71.9) | 412 (28.1) | 21.6% (11.9–31.2) | <0.0001 | 0.4% (-6.5–7.3) | 0.9051 |
| Main occupation |  |  |  | <0.0001^$^ |  | 0.0002^$^ |
| Self-employment^2^ | 3214 (54.4) | 2693 (45.6) | 0 | – | 0 | – |
| Private paid work | 409 (56.8) | 311 (43.2) | 4.1% (-1.9–10.0) | 0.1268 | -1.5% (-5.5–2.6) | 0.4205 |
| Government paid work | 499 (78.5) | 137 (21.5) | 20.9% (14.6–27.2) | <0.0001 | 9.0% (4.0–14.0) | 0.0001 |
| Others^3^ | 1808 (50.8) | 1754 (49.2) | 0.5% (-4.1–5.0) | 0.8226 | 3.8% (-1.9–9.6) | 0.1360 |
| Residence |  |  |  |  |  |  |
| Rural | 4673 (55.5) | 3743 (44.5) | 0 | – | 0 | – |
| Urban or semi-urban | 1257 (52.2) | 1152 (47.8) | -3.2% (-19.0–12.6) | 0.6488 | -3.1% (-18.2–12.0) | 0.6429 |
| Usual monthly income, NGN |  |  |  | 0.0001^$^ |  | 0.0028^$^ |
| No income | 1297 (43.5) | 1683 (56.5) | 0 | – | 0 | – |
| 20,000 and less | 2651 (58.9) | 1849 (41.1) | 6.0% (1.3–10.8) | 0.0041 | 8.1% (1.9–14.3) | 0.0034 |
| More than 20,000 | 1982 (59.3) | 1363 (40.7) | 10.6% (4.9–16.2) | <0.0001 | 9.8% (3.4–16.4) | 0.0007 |
| Main source of information about COVID-19 |  |  |  | 0.7471^$^ |  | 0.5652^$^ |
| Internet, social media (whatsapp, facebook), & SMS | 447 (55.0) | 366 (45.0) | 0 | – | 0 | – |
| Traditional media (television, radio, prints) | 3042 (50.6) | 2975 (49.4) | -0.6% (-7.5–6.4) | 0.8426 | -1.4% (-9.4–6.6) | 0.6976 |
| Interpersonal^4^ | 2441 (61.1) | 1554 (38.9) | -2.8% (-11.1–5.6) | 0.4592 | -3.4% (-12.7–5.9) | 0.4131 |
| Most trusted source of information about COVID-19 |  |  |  | 0.8047^$^ |  | 0.1897^$^ |
| Internet, social media (whatsapp, facebook), & SMS | 357 (55.1) | 291 (44.9) | 0 | – | 0 | – |
| Traditional media (television, radio, prints) | 2983 (50.7) | 2900 (49.3) | 1.8% (-4.8–8.5) | 0.5340 | 3.9% (-6.0–13.8) | 0.3754 |
| Interpersonal^4^ | 2590 (60.3) | 1704 (39.7) | 1.5% (-6.4–9.5) | 0.6649 | 7.2% (-3.9–18.4) | 0.1467 |
| Level of knowledge about COVID-19^5^ |  |  |  |  |  |  |
| Poor | 4981 (51.2) | 4750 (48.8) | 0 | – | 0 | – |
| Good | 949 (86.8) | 145 (13.2) | 22.2% (14.9–29.6) | <0.0001 | 10.7% (4.2–17.2) | 0.0002 |
| Level of attitude towards COVID-19 (vaccination)^6^ |  |  |  |  |  |  |
| Poor | 1630 (31.0) | 3630 (69.0) | 0 | – | 0 | – |
| Good | 4300 (77.3) | 1265 (22.7) | 42.4% (33.4–51.5) | <0.0001 | 40.3% (31.6–49.0) | <0.0001 |
| ^Very important or important. ^^Not important at all, not important, or not sure. cPD=Crude prevalence difference. aPD=Adjusted prevalence difference. cCoef=Crude coefficient. aCoef=Adjusted coefficient. *Adjusted for clustering. **Adjusted for clustering; Basic knowledge of COVID-19; Attitude towards COVID-19 & COVID-19 vaccination; Source of information about COVID-19 (Main source and Most trusted source of information about COVID-19); and Sociodemographic characteristics (Gender, Age, Marital status, Educational level, Occupation, Residence (rural vs urban or semi-urban), and Monthly income). ^$^p value of overall effect. ^1^Separated or Divorced or Widowed or Never married (Single). ^2^Farmer or Trader or Other self-employments. ^3^Housewife or Student or Apprentice or Youth Corper or None. ^4^Relatives/friends, health workers, place of work, place of worship etc. ^5^Knowledge score of <75% of the highest attainable score of 44 was poor knowledge and >=75% was good knowledge. ^6^Attitude score of <75% of the highest attainable score of 80 was poor attitude and >=75% was good attitude. | | | | | | |

The perceived importance of receiving COVID-19 vaccination and the associations between it and sociodemographic and background factors are presented in table 3. Among the 10825 study participants, 5930 (54.8%) said it was important for them to receive COVID-19 vaccination while 4895 (45.2%) said it was not important or that they were not sure about it. The predictors of having the perception that it was important to receive COVID-19 vaccination were: good attitude towards COVID-19 (vaccination) (aPD 40.3%, 31.6–49.0, p<0.0001); good knowledge about COVID-19 (aPD 10.7%, 4.2–17.2, p=0.0002); female gender (aPD 2.4%, 0.2–4.6, p=0.0129); main occupation (adjusted p value of overall effect=0.0002); and level of monthly income (adjusted p value of overall effect=0.0028).

| Table 4: Association between sociodemographic and background factors and the fear of having severe side-effects from COVID-19 vaccination among the 10825 study participants | | | | | | |
| --- | --- | --- | --- | --- | --- | --- |
|  | **Fear of having severe side-effects from**  **COVID-19 vaccination** | | **Crude results*** | | **Adjusted results**** | |
|  | **Not fearful^**  **n (%)**  **4439 (41.0)** | **Fearful/not sure^^**  **n (%)**  **6386 (59.0)** | cPD (97.5% CI) or  cCoef (97.5% CI) | p value | aPD (97.5% CI) or  aCoef (97.5% CI) | p value |
| Gender |  |  |  |  |  |  |
| Female | 2431 (40.0) | 3645 (60.0) | 0 | – | 0 | – |
| Male | 2008 (42.3) | 2741 (57.7) | 3.0% (0.6–5.5) | 0.0054 | 1.9% (-0.8–4.6) | 0.1097 |
| Age, years (coefficient) | – | – | 0.04% (-0.1–0.2) | 0.4348 | -0.1% (-0.2–0.1) | 0.2770 |
| Marital status |  |  |  |  |  |  |
| Not married^1^ | 1934 (37.8) | 3179 (62.2) | 0 | – | 0 | – |
| Married | 2505 (43.9) | 3207 (56.1) | 4.2% (0.7–7.7) | 0.0074 | 1.7% (-1.4–4.9) | 0.2145 |
| Educational level |  |  |  | 0.0007^$^ |  | 0.0818^$^ |
| No formal education | 444 (41.7) | 621 (58.3) | 0 | – | 0 | – |
| Primary | 863 (39.0) | 1348 (61.0) | 3.3% (-1.3–7.8) | 0.1080 | -0.9% (-5.8–3.9) | 0.6667 |
| Secondary | 2287 (37.6) | 3796 (62.4) | 2.8% (-2.0–7.5) | 0.1919 | -3.2% (-8.2–1.9) | 0.1643 |
| Tertiary | 845 (57.6) | 621 (42.4) | 14.8% (6.3–23.4) | 0.0001 | 2.7% (-5.0–10.4) | 0.4373 |
| Main occupation |  |  |  | <0.0001^$^ |  | 0.1694^$^ |
| Self-employment^2^ | 2453 (41.5) | 3454 (58.5) | 0 | – | 0 | – |
| Private paid work | 311 (43.2) | 409 (56.8) | 3.0% (-1.8–7.9) | 0.1605 | -0.4% (-4.0–3.3) | 0.8097 |
| Government paid work | 407 (64.0) | 229 (36.0) | 15.0% (7.1–22.8) | <0.0001 | 6.5% (-7.4–13.7) | 0.0442 |
| Others^3^ | 1268 (35.6) | 2294 (64.4) | -3.2% (-6.7–0.2) | 0.0374 | -0.03% (-4.6–4.6) | 0.9877 |
| Residence |  |  |  |  |  |  |
| Urban or semi-urban | 933 (38.7) | 1476 (61.3) | 0 | – | 0 | – |
| Rural | 3506 (41.7) | 4910 (58.3) | 3.5% (-8.5–15.4) | 0.5183 | 3.1% (-8.9–15.0) | 0.5658 |
|  |  |  |  |  |  |  |
| Usual monthly income, NGN |  |  |  | <0.0001^$^ |  | 0.0013^$^ |
| No income | 1001 (33.6) | 1979 (66.4) | 0 | – | 0 | – |
| 20,000 and less | 1859 (41.3) | 2641 (58.7) | 5.3% (1.4–9.3) | 0.0024 | 4.6% (0.2–9.0) | 0.0187 |
| More than 20,000 | 1579 (47.2) | 1766 (52.8) | 11.0% (6.3–15.7) | <0.0001 | 7.1% (2.7–11.5) | 0.0003 |
| Main source of information about COVID-19 |  |  |  | 0.1427^$^ |  | 0.7444^$^ |
| Internet, social media (whatsapp, facebook), & SMS | 332 (40.8) | 481 (59.2) | 0 | – | 0 | – |
| Traditional media (television, radio, prints) | 2213 (36.8) | 3804 (63.2) | -4.5% (-10.1–1.2) | 0.0758 | -2.9% (-11.8–6.0) | 0.4623 |
| Interpersonal^4^ | 1894 (47.4) | 2101 (52.6) | -5.2% (-12.0–1.7) | 0.0895 | -1.7% (-10.2–6.7) | 0.6430 |
| Most trusted source of information about COVID-19 |  |  |  | 0.1357^$^ |  | 0.7639^$^ |
| Internet, social media (whatsapp, facebook), & SMS | 275 (42.4) | 373 (57.6) | 0 | – | 0 | – |
| Traditional media (television, radio, prints) | 2158 (36.7) | 3725 (63.3) | -4.1% (-9.5–1.3) | 0.0855 | -2.5% (-10.8–5.8) | 0.5023 |
| Interpersonal^4^ | 2006 (46.7) | 2288 (53.3) | -5.1% (-11.6–1.5) | 0.0836 | -2.5% (-10.4–5.5) | 0.4860 |
| Level of knowledge about COVID-19^5^ |  |  |  |  |  |  |
| Poor | 3781 (38.9) | 5950 (61.1) | 0 | – | 0 | – |
| Good | 658 (60.2) | 436 (39.8) | 14.2% (5.3–23.1) | 0.0003 | 9.3% (0.7–17.9) | 0.0155 |
| Level of attitude towards COVID-19 (vaccination)^6^ |  |  |  |  |  |  |
| Poor | 1580 (30.0) | 3680 (70.0) | 0 | – | 0 | – |
| Good | 2859 (51.4) | 2706 (48.6) | 18.6% (8.5–28.7) | <0.0001 | 16.9% (7.1–26.7) | 0.0001 |
| ^Not fearful at all or not fearful. ^^Very fearful, a little fearful, or not sure. cPD=Crude prevalence difference. aPD=Adjusted prevalence difference. cCoef=Crude coefficient. aCoef=Adjusted coefficient. *Adjusted for clustering. **Adjusted for clustering; Basic knowledge of COVID-19; Attitude towards COVID-19 & COVID-19 vaccination; Source of information about COVID-19 (Main source and Most trusted source of information about COVID-19); and Sociodemographic characteristics (Gender, Age, Marital status, Educational level, Occupation, Residence (rural vs urban or semi-urban), and Monthly income). ^$^p value of overall effect. ^1^Separated or Divorced or Widowed or Never married (Single). ^2^Farmer or Trader or Other self-employments. ^3^Housewife or Student or Apprentice or Youth Corper or None. ^4^Relatives/friends, health workers, place of work, place of worship etc. ^5^Knowledge score of <75% of the highest attainable score of 44 was poor knowledge and >=75% was good knowledge. ^6^Attitude score of <75% of the highest attainable score of 80 was poor attitude and >=75% was good attitude. | | | | | | |

The fear of having severe side-effects from COVID-19 vaccination and the associations between it and sociodemographic and background factors are presented in table 4. Among the 10825 study participants, 4439 (41.0%) were not fearful of having severe side-effects from COVID-19 vaccination while 6386 (59.0%) were fearful or not sure about it. The predictors of not being fearful of having severe side-effects from COVID-19 vaccination were: good attitude towards COVID-19 (vaccination) (aPD 16.9%, 7.1–26.7, p=0.0001); good knowledge about COVID-19 (aPD 9.3%, 0.7–17.9, p=0.0155); and level of monthly income (adjusted p value of overall effect=0.0013).

| Table 5: Association between sociodemographic and background factors and the perceived protection against COVID-19 from receiving COVID-19 vaccination among the 10825 study participants | | | | | | |
| --- | --- | --- | --- | --- | --- | --- |
|  | **Perceived protection from receiving COVID-19 vaccination** | | **Crude results*** | | **Adjusted results**** | |
|  | **Protection^**  **n (%)**  **5580 (51.6)** | **No protection/ not sure^^**  **n (%)**  **5245 (48.4)** | cPD (97.5% CI) or  cCoef (97.5% CI) | p value | aPD (97.5% CI) or  aCoef (97.5% CI) | p value |
| Gender |  |  |  |  |  |  |
| Male | 2414 (50.8) | 2335 (49.2) | 0 | – | 0 | – |
| Female | 3166 (52.1) | 2910 (47.9) | 0.7% (-2.4–3.7) | 0.6217 | 1.1% (-1.3–3.5) | 0.2967 |
| Age, years (coefficient) | – | – | -0.1% (-0.3–0.01) | 0.0425 | -0.2% (-0.4–(-0.02) | 0.0154 |
| Marital status |  |  |  |  |  |  |
| Not married^1^ | 2503 (48.9) | 2610 (51.1) | 0 | – | 0 | – |
| Married | 3077 (53.9) | 2635 (46.1) | 3.6% (-0.4–7.6) | 0.0455 | 4.3% (0.9–7.7) | 0.0049 |
| Educational level |  |  |  | <0.0001^$^ |  | 0.0226^$^ |
| No formal education | 537 (50.4) | 528 (49.6) | 0 | – | 0 | – |
| Primary | 900 (40.7) | 1311 (59.3) | 1.4% (-6.2–9.0) | 0.6781 | -6.8% (-12.6–(-1.0) | 0.0087 |
| Secondary | 3100 (51.0) | 2983 (49.0) | 7.6% (-0.02–15.2) | 0.0255 | -5.1% (-12.9–2.6) | 0.1372 |
| Tertiary | 1043 (71.2) | 423 (28.8) | 22.3% (12.5–32.1) | <0.0001 | 2.4% (-7.3–12.0) | 0.5839 |
| Main occupation |  |  |  | <0.0001^$^ |  | 0.0338^$^ |
| Self-employment^2^ | 2970 (50.3) | 2937 (49.7) | 0 | – | 0 | – |
| Private paid work | 375 (52.1) | 345 (47.9) | 2.5% (-3.6–8.5) | 0.3556 | -3.9% (-9.1–1.3) | 0.0920 |
| Government paid work | 487 (76.6) | 149 (23.4) | 19.3% (12.0–26.7) | <0.0001 | 5.7% (-1.7–13.1) | 0.0822 |
| Others^3^ | 1748 (49.1) | 1814 (50.9) | 0.8% (-3.6–5.3) | 0.6756 | 3.1% (-4.1–10.2) | 0.3329 |
| Residence |  |  |  |  |  |  |
| Rural | 4334 (51.5) | 4082 (48.5) | 0 | – | 0 | – |
| Urban or semi-urban | 1246 (51.7) | 1163 (48.3) | -0.1% (-15.6–15.5) | 0.9894 | -1.0% (-16.6–14.7) | 0.8909 |
| Usual monthly income, NGN |  |  |  | 0.0002^$^ |  | 0.0385^$^ |
| No income | 1312 (44.0) | 1668 (56.0) | 0 | – | 0 | – |
| 20,000 and less | 2286 (50.8) | 2214 (49.2) | 4.5% (-1.3–10.4) | 0.0834 | 6.7% (-2.1–15.5) | 0.0891 |
| More than 20,000 | 1982 (59.3) | 1363 (40.7) | 10.5% (4.5–16.5) | 0.0001 | 10.0% (1.2–18.9) | 0.0112 |
| Main source of information about COVID-19 |  |  |  | 0.7337^$^ |  | 0.7686^$^ |
| Internet, social media (whatsapp, facebook), & SMS | 440 (54.1) | 373 (45.9) | 0 | – | 0 | – |
| Traditional media (television, radio, prints) | 2774 (46.1) | 3243 (53.9) | -3.4% (-13.0–6.3) | 0.4343 | 2.4% (-7.6–12.4) | 0.5877 |
| Interpersonal^4^ | 2366 (59.2) | 1629 (40.8) | -2.7% (-14.3–9.0) | 0.6093 | 1.5% (-11.0–14.0) | 0.7919 |
| Most trusted source of information about COVID-19 |  |  |  | 0.5963^$^ |  | 0.0867^$^ |
| Internet, social media (whatsapp, facebook), & SMS | 368 (56.8) | 280 (43.2) | 0 | – | 0 | – |
| Traditional media (television, radio, prints) | 2575 (43.8) | 3308 (56.2) | -4.1% (-14.3–6.1) | 0.3680 | -4.7% (-15.8–6.4) | 0.3425 |
| Interpersonal^4^ | 2637 (61.4) | 1657 (38.6) | -0.8% (-10.6–8.9) | 0.8491 | 1.6% (-7.0–10.3) | 0.6709 |
| Level of knowledge about COVID-19^5^ |  |  |  |  |  |  |
| Poor | 4945 (50.8) | 4786 (49.2) | 0 | – | 0 | – |
| Good | 635 (58.0) | 459 (42.0) | 14.0% (-7.3–35.3) | 0.1418 | 3.9% (-13.4–21.3) | 0.6116 |
| Level of attitude towards COVID-19 (vaccination)^6^ |  |  |  |  |  |  |
| Poor | 1709 (32.5) | 3551 (67.5) | 0 | – | 0 | – |
| Good | 3871 (69.6) | 1694 (30.4) | 40.5% (30.5–50.5) | <0.0001 | 38.8% (29.1–48.4) | <0.0001 |
| ^Full protection or partial protection. ^^No protection at all, no protection, or not sure. cPD=Crude prevalence difference. aPD=Adjusted prevalence difference. cCoef=Crude coefficient. aCoef=Adjusted coefficient. *Adjusted for clustering. **Adjusted for clustering; Basic knowledge of COVID-19; Attitude towards COVID-19 & COVID-19 vaccination; Source of information about COVID-19 (Main source and Most trusted source of information about COVID-19); and Sociodemographic characteristics (Gender, Age, Marital status, Educational level, Occupation, Residence (rural vs urban or semi-urban), and Monthly income). ^$^p value of overall effect. ^1^Separated or Divorced or Widowed or Never married (Single). ^2^Farmer or Trader or Other self-employments. ^3^Housewife or Student or Apprentice or Youth Corper or None. ^4^Relatives/friends, health workers, place of work, place of worship etc. ^5^Knowledge score of <75% of the highest attainable score of 44 was poor knowledge and >=75% was good knowledge. ^6^Attitude score of <75% of the highest attainable score of 80 was poor attitude and >=75% was good attitude. | | | | | | |

The perceived protection against COVID-19 from receiving COVID-19 vaccination and the associations between it and sociodemographic and background factors are presented in table 5. Among the 10825 study participants, 5580 (51.6%) said COVID-19 vaccination would give them protection against COVID-19 while 5245 (48.4%) said it would give no protection or that they were not sure about it. The predictors of having the perception that COVID-19 vaccination would give protection against COVID-19 were: good attitude towards COVID-19 (vaccination) (aPD 38.8%, 29.1–48.4, p<0.0001); being married (aPD 4.3%, 0.9–7.7, p=0.0049); educational level (adjusted p value of overall effect=0.0226); and age as one year increase in age reduces the probability of perceiving that COVID-19 vaccination would give protection by 0.2% (aCoef -0.2%, -0.4–(-0.02), p=0.0154).

| Table 6: Association between sociodemographic and background factors and the awareness of the availability of COVID-19 vaccination for receipt among the 10825 study participants | | | | | | |
| --- | --- | --- | --- | --- | --- | --- |
|  | **Ever heard COVID-19 vaccination was available for receipt** | | **Crude results*** | | **Adjusted results**** | |
|  | **Yes**  **n (%)**  **7922 (73.2)** | **No/not sure^^**  **n (%)**  **2903 (26.8)** | cPD (97.5% CI) or  cCoef (97.5% CI) | p value | aPD (97.5% CI) or  aCoef (97.5% CI) | p value |
| Gender |  |  |  |  |  |  |
| Male | 3537 (74.5) | 1212 (25.5) | 0 | – | 0 | – |
| Female | 4385 (72.2) | 1691 (27.8) | -1.8% (-4.1–0.5) | 0.0746 | -0.9% (-3.2–1.5) | 0.4106 |
| Age, years (coefficient) | – | – | -0.1% (-0.3–(-0.01) | 0.0172 | -0.2% (-0.4–0.02) | 0.0402 |
| Marital status |  |  |  |  |  |  |
| Not married^1^ | 3653 (71.5) | 1460 (28.5) | 0 | – | 0 | – |
| Married | 4269 (74.7) | 1443 (25.3) | 2.9% (0.3–5.5) | 0.0122 | 4.2% (0.8–7.6) | 0.0056 |
| Educational level |  |  |  | <0.0001^$^ |  | 0.0020^$^ |
| No formal education | 708 (66.5) | 357 (33.5) | 0 | – | 0 | – |
| Primary | 1491 (67.4) | 720 (32.6) | 8.1% (2.2–14.0) | 0.0020 | 3.2% (-4.2–10.5) | 0.3324 |
| Secondary | 4348 (71.5) | 1735 (28.5) | 11.5% (5.1–17.8) | <0.0001 | 4.1% (-2.9–11.1) | 0.1905 |
| Tertiary | 1375 (93.8) | 91 (6.2) | 23.7% (15.5–31.9) | <0.0001 | 10.8% (3.1–18.5) | 0.0016 |
| Main occupation |  |  |  | <0.0001^$^ |  | 0.4406^$^ |
| Self-employment^2^ | 4182 (70.8) | 1725 (29.2) | 0 | – | 0 | – |
| Private paid work | 595 (82.6) | 125 (17.4) | 7.3% (2.4–12.2) | 0.0009 | 1.9% (-2.8–6.5) | 0.3640 |
| Government paid work | 609 (95.6) | 27 (4.2) | 15.6% (9.1–22.1) | <0.0001 | 3.9% (-1.4–9.2) | 0.1022 |
| Others^3^ | 2536 (71.2) | 1026 (28.8) | 0.8% (-2.2–3.7) | 0.5565 | 1.7% (-3.2–6.7) | 0.4385 |
| Residence |  |  |  |  |  |  |
| Rural | 5959 (70.8) | 2457 (29.2) | 0 | – | 0 | – |
| Urban or semi-urban | 1963 (81.5) | 446 (18.5) | 10.4% (-7.7–28.6) | 0.1985 | 8.5% (-8.9–25.9) | 0.2729 |
| Usual monthly income, NGN |  |  |  | 0.0004^$^ |  | 0.0956^$^ |
| No income | 2008 (67.4) | 972 (32.6) | 0 | – | 0 | – |
| 20,000 and less | 3197 (71.0) | 1303 (29.0) | 2.5% (-0.6–5.5) | 0.0672 | 3.9% (-0.9–8.7) | 0.0682 |
| More than 20,000 | 2717 (81.2) | 628 (18.8) | 8.6% (3.5–13.7) | 0.0001 | 7.3% (-0.5–15.2) | 0.0369 |
| Main source of information about COVID-19 |  |  |  | 0.1105^$^ |  | 0.2123^$^ |
| Internet, social media (whatsapp, facebook), & SMS | 599 (73.7) | 214 (26.3) | 0 | – | 0 | – |
| Traditional media (television, radio, prints) | 4151 (69.0) | 1866 (31.0) | -7.1% (-14.9–0.6) | 0.0399 | -8.9% (-20.7–2.9) | 0.0905 |
| Interpersonal^4^ | 3172 (79.4) | 823 (20.6) | -6.0% (-15.0–2.9) | 0.1310 | -8.6% (-20.3–3.1) | 0.1010 |
| Most trusted source of information about COVID-19 |  |  |  | 0.3643^$^ |  | 0.3706^$^ |
| Internet, social media (whatsapp, facebook), & SMS | 481 (74.2) | 167 (25.8) | 0 | – | 0 | – |
| Traditional media (television, radio, prints) | 4157 (70.7) | 1726 (29.3) | -5.2% (-13.4–3.0) | 0.1582 | 3.5% (-8.9–15.9) | 0.5296 |
| Interpersonal^4^ | 3284 (76.5) | 1010 (23.5) | -3.0% (-12.1–6.2) | 0.4655 | 7.2% (-5.6–20.0) | 0.2061 |
| Level of knowledge about COVID-19^5^ |  |  |  |  |  |  |
| Poor | 6975 (71.7) | 2756 (28.3) | 0 | – | 0 | – |
| Good | 947 (86.6) | 147 (13.4) | 5.8% (-7.4–19.0) | 0.3271 | 0.9% (-10.0–11.8) | 0.8603 |
| Level of attitude towards COVID-19 (vaccination)^6^ |  |  |  |  |  |  |
| Poor | 3534 (67.2) | 1726 (32.8) | 0 | – | 0 | – |
| Good | 4388 (78.9) | 1177 (21.1) | 9.9% (2.7–17.2) | 0.0021 | 8.1% (1.3–15.0) | 0.0079 |
| ^Heard many times or few times or once. ^^Heard no time at all, no time, or not sure. cPD=Crude prevalence difference. aPD=Adjusted prevalence difference. cCoef=Crude coefficient. aCoef=Adjusted coefficient. *Adjusted for clustering. **Adjusted for clustering; Basic knowledge of COVID-19; Attitude towards COVID-19 & COVID-19 vaccination; Source of information about COVID-19 (Main source and Most trusted source of information about COVID-19); and Sociodemographic characteristics (Gender, Age, Marital status, Educational level, Occupation, Residence (rural vs urban or semi-urban), and Monthly income). ^$^p value of overall effect. ^1^Separated or Divorced or Widowed or Never married (Single). ^2^Farmer or Trader or Other self-employments. ^3^Housewife or Student or Apprentice or Youth Corper or None. ^4^Relatives/friends, health workers, place of work, place of worship etc. ^5^Knowledge score of <75% of the highest attainable score of 44 was poor knowledge and >=75% was good knowledge. ^6^Attitude score of <75% of the highest attainable score of 80 was poor attitude and >=75% was good attitude. | | | | | | |

The awareness of the availability of COVID-19 vaccination for receipt and the associations between it and sociodemographic and background factors are presented in table 6. Among the 10825 study participants, 7922 (73.2%) had ever heard COVID-19 vaccination was available for them to go and receive while 2903 (26.8%) had not heard or were not sure about it. The predictors of being aware COVID-19 vaccination was available for receipt were: good attitude towards COVID-19 (vaccination) (aPD 8.1%, 1.3–15.0, p=0.0079); being married (aPD 4.2%, 0.8–7.6, p=0.0056); and educational level (adjusted p value of overall effect=0.0020).

| Table 7: Association between sociodemographic and background factors and the knowledge of COVID-19 vaccination place among the 10825 study participants | | | | | | |
| --- | --- | --- | --- | --- | --- | --- |
|  | **Knowledge of COVID-19 vaccination place** | | **Crude results*** | | **Adjusted results**** | |
|  | **Knew a close place^**  **n (%)**  **5136 (47.5)** | **Knew no place/ far place^^**  **n (%)**  **5689 (52.5)** | cPD (97.5% CI) or  cCoef (97.5% CI) | p value | aPD (97.5% CI) or  aCoef (97.5% CI) | p value |
| Gender |  |  |  |  |  |  |
| Male | 2272 (47.8) | 2477 (52.2) | 0 | – | 0 | – |
| Female | 2864 (47.1) | 3212 (52.9) | -0.1% (-2.3–2.0) | 0.8782 | 0.04% (-2.1–2.2) | 0.9654 |
| Age, years (coefficient) | – | – | -0.04% (-0.2–0.1) | 0.4919 | -0.2% (-0.4–(-0.1) | 0.0032 |
| Marital status |  |  |  |  |  |  |
| Not married^1^ | 2240 (43.8) | 2873 (56.2) | 0 | – | 0 | – |
| Married | 2896 (50.7) | 2816 (49.3) | 6.5% (3.2–9.9) | <0.0001 | 6.2% (3.2–9.1) | <0.0001 |
| Educational level |  |  |  | <0.0001^$^ |  | 0.0327^$^ |
| No formal education | 485 (45.5) | 580 (54.5) | 0 | – | 0 | – |
| Primary | 974 (44.1) | 1237 (55.9) | 4.0% (-1.8–9.8) | 0.1244 | -1.9% (-7.8–4.1) | 0.4819 |
| Secondary | 2646 (43.5) | 3437 (56.5) | 3.9% (-1.6–9.6) | 0.1141 | -3.2% (-10.7–4.4) | 0.3482 |
| Tertiary | 1031 (70.3) | 435 (29.7) | 15.4% (8.5–22.4) | <0.0001 | 3.6% (-5.0–12.1) | 0.3519 |
| Main occupation |  |  |  | <0.0001^$^ |  | 0.1660^$^ |
| Self-employment^2^ | 2727 (46.2) | 3180 (53.8) | 0 | – | 0 | – |
| Private paid work | 410 (56.9) | 310 (43.1) | 4.4% (-1.2–9.9) | 0.0789 | 0.5% (-5.0–6.0) | 0.8509 |
| Government paid work | 475 (74.7) | 161 (25.3) | 13.9% (8.7–19.1) | <0.0001 | 5.2% (-0.04-10.5) | 0.0261 |
| Others^3^ | 1524 (42.8) | 2038 (57.2) | -3.0% (-7.0–1.0) | 0.0980 | 0.9% (-4.7–6.6) | 0.7163 |
| Residence |  |  |  |  |  |  |
| Rural | 3863 (45.9) | 4553 (54.1) | 0 | – | 0 | – |
| Urban or semi-urban | 1273 (52.8) | 1136 (47.2) | 6.6% (-21.9–35.0) | 0.6047 | 5.6% (-21.6–32.7) | 0.6453 |
| Usual monthly income, NGN |  |  |  | 0.0002^$^ |  | 0.1323^$^ |
| No income | 1209 (40.6) | 1771 (59.4) | 0 | – | 0 | – |
| 20,000 and less | 2063 (45.8) | 2437 (54.2) | 5.1% (1.1–9.1) | 0.0040 | 4.7% (-1.8–11.3) | 0.1054 |
| More than 20,000 | 1864 (55.7) | 1481 (44.3) | 10.9% (4.9–17.0) | 0.0001 | 8.3% (-1.0–17.7) | 0.0458 |
| Main source of information about COVID-19 |  |  |  | 0.0178^$^ |  | 0.2182^$^ |
| Internet, social media (whatsapp, facebook), & SMS | 273 (33.6) | 540 (66.4) | 0 | – | 0 | – |
| Traditional media (television, radio, prints) | 2512 (41.8) | 3505 (58.2) | 4.2% (-3.0–11.4) | 0.1912 | 1.9% (-4.8–8.6) | 0.5315 |
| Interpersonal^4^ | 2351 (58.9) | 1644 (41.1) | 7.6% (1.6–13.6) | 0.0046 | 4.5% (-1.5–10.4) | 0.0904 |
| Most trusted source of information about COVID-19 |  |  |  | 0.0086^$^ |  | 0.0575^$^ |
| Internet, social media (whatsapp, facebook), & SMS | 232 (35.8) | 416 (64.2) | 0 | – | 0 | – |
| Traditional media (television, radio, prints) | 2538 (43.1) | 3345 (56.9) | 5.3% (-1.9–12.4) | 0.0986 | 3.0% (-2.3–8.2) | 0.2003 |
| Interpersonal^4^ | 2366 (55.1) | 1928 (44.9) | 9.0% (2.5–15.6) | 0.0021 | 6.5% (0.4–12.6) | 0.0175 |
| Level of knowledge about COVID-19^5^ |  |  |  |  |  |  |
| Poor | 4321 (44.4) | 5410 (55.6) | 0 | – | 0 | – |
| Good | 815 (74.5) | 279 (25.5) | 11.9% (1.0–22.8) | 0.0147 | 7.3% (-3.5–18.1) | 0.1293 |
| Level of attitude towards COVID-19 (vaccination)^6^ |  |  |  |  |  |  |
| Poor | 2073 (39.4) | 3187 (60.6) | 0 | – | 0 | – |
| Good | 3063 (55.0) | 2502 (45.0) | 12.7% (5.4–19.9) | 0.0001 | 11.0% (3.8–18.2) | 0.0006 |
| ^Knew a very close place or a close place. ^^Knew no place, a very far place, or a far place. cPD=Crude prevalence difference. aPD=Adjusted prevalence difference. cCoef=Crude coefficient. aCoef=Adjusted coefficient. *Adjusted for clustering. **Adjusted for clustering; Basic knowledge of COVID-19; Attitude towards COVID-19 & COVID-19 vaccination; Source of information about COVID-19 (Main source and Most trusted source of information about COVID-19); and Sociodemographic characteristics (Gender, Age, Marital status, Educational level, Occupation, Residence (rural vs urban or semi-urban), and Monthly income). ^$^p value of overall effect. ^1^Separated or Divorced or Widowed or Never married (Single). ^2^Farmer or Trader or Other self-employments. ^3^Housewife or Student or Apprentice or Youth Corper or None. ^4^Relatives/friends, health workers, place of work, place of worship etc. ^5^Knowledge score of <75% of the highest attainable score of 44 was poor knowledge and >=75% was good knowledge. ^6^Attitude score of <75% of the highest attainable score of 80 was poor attitude and >=75% was good attitude. | | | | | | |

The knowledge of COVID-19 vaccination place and the associations between it and sociodemographic and background factors are presented in table 7. Among the 10825 study participants, 5136 (47.5%) knew a close COVID-19 vaccination place while 5689 (52.5%) knew a far place or no place. The predictors of knowing a close COVID-19 vaccination place were: good attitude towards COVID-19 (vaccination) (aPD 11.0%, 3.8–18.2, p=0.0006); being married (aPD 6.2%, 3.2–9.1, p<0.0001); and age as one year increase in age reduces the probability of knowing a close COVID-19 vaccination place by 0.2% (aCoef -0.2%, -0.4–(-0.1), p=0.0032).
